# Nogo receptor-Fc delivered by haematopoietic cells enhances neurorepair in a multiple sclerosis model

**DOI:** 10.1093/braincomms/fcad108

**Published:** 2023-04-04

**Authors:** Sining Ye, Paschalis Theotokis, Jae Young Lee, Min Joung Kim, Danica Nheu, Olivia Ellen, Thomas Bedford, Padmanabhan Ramanujam, David K Wright, Stuart J McDonald, Amani Alrehaili, Maha Bakhuraysah, Jung Hee Kang, Christopher Siatskas, Cedric S Tremblay, David J Curtis, Nikolaos Grigoriadis, Mastura Monif, Stephen M Strittmatter, Steven Petratos

**Affiliations:** Department of Neuroscience, Central Clinical School, Monash University, Prahran, Victoria 3004, Australia; B’, Department of Neurology, Laboratory of Experimental Neurology and Neuroimmunology, AHEPA University Hospital, Stilponos Kiriakides str. 1, 54636 Thessaloniki, Macedonia, Greece; ToolGen Inc., Gangseo-gu, 07789 Seoul, Korea; Department of Neuroscience, Central Clinical School, Monash University, Prahran, Victoria 3004, Australia; Department of Neuroscience, Central Clinical School, Monash University, Prahran, Victoria 3004, Australia; Department of Neuroscience, Central Clinical School, Monash University, Prahran, Victoria 3004, Australia; Department of Neuroscience, Central Clinical School, Monash University, Prahran, Victoria 3004, Australia; Department of Neuroscience, Central Clinical School, Monash University, Prahran, Victoria 3004, Australia; Department of Neuroscience, Central Clinical School, Monash University, Prahran, Victoria 3004, Australia; Department of Neuroscience, Central Clinical School, Monash University, Prahran, Victoria 3004, Australia; Department of Neuroscience, Central Clinical School, Monash University, Prahran, Victoria 3004, Australia; Department of Clinical Laboratory Sciences, College of Applied Medical Sciences, Taif University, PO Box 11099, Taif 21944, Saudi Arabia; Department of Neuroscience, Central Clinical School, Monash University, Prahran, Victoria 3004, Australia; Department of Clinical Laboratory Sciences, College of Applied Medical Sciences, Taif University, PO Box 11099, Taif 21944, Saudi Arabia; Department of Neuroscience, Central Clinical School, Monash University, Prahran, Victoria 3004, Australia; STEMCELL Technologies Inc., Vancouver, British Columbia V6A 1B6, Canada; Australian Centre for Blood Diseases, Central Clinical School, Monash University, Prahran, Victoria 3004, Australia; Australian Centre for Blood Diseases, Central Clinical School, Monash University, Prahran, Victoria 3004, Australia; Clinical Haematology, Alfred Hospital, Prahran, Victoria 3004, Australia; B’, Department of Neurology, Laboratory of Experimental Neurology and Neuroimmunology, AHEPA University Hospital, Stilponos Kiriakides str. 1, 54636 Thessaloniki, Macedonia, Greece; Department of Neuroscience, Central Clinical School, Monash University, Prahran, Victoria 3004, Australia; Program in Cellular Neuroscience, Neurodegeneration and Repair, Yale University School of Medicine, New Haven, CT 06536, USA; Department of Neuroscience, Central Clinical School, Monash University, Prahran, Victoria 3004, Australia

**Keywords:** Nogo receptor-Fc, haematopoietic stem cells, experimental autoimmune encephalomyelitis, remyelination, axonal regeneration

## Abstract

Nogo receptor 1 is the high affinity receptor for the potent myelin-associated inhibitory factors that make up part of the inflammatory extracellular milieu during experimental autoimmune encephalomyelitis. Signalling through the Nogo receptor 1 complex has been shown to be associated with axonal degeneration in an animal model of multiple sclerosis, and neuronal deletion of this receptor homologue, in a disease specific manner, is associated with preserving axons even in the context of neuroinflammation. The local delivery of Nogo receptor(1-310)-Fc, a therapeutic fusion protein, has been successfully applied as a treatment in animal models of spinal cord injury and glaucoma. As multiple sclerosis and experimental autoimmune encephalomyelitis exhibit large numbers of inflammatory cell infiltrates within the CNS lesions, we utilized transplantable haematopoietic stem cells as a cellular delivery method of the Nogo receptor(1-310)-Fc fusion protein. We identified CNS-infiltrating macrophages as the predominant immune-positive cell type that overexpressed myc-tagged Nogo receptor(1-310)-Fc fusion protein at the peak stage of experimental autoimmune encephalomyelitis. These differentiated phagocytes were predominant during the extensive demyelination and axonal damage, which are associated with the engulfment of the protein complex of Nogo receptor(1-310)-Fc binding to myelin ligands. Importantly, mice transplanted with haematopoietic stem cells transduced with the lentiviral vector carrying Nogo receptor(1-310)-Fc and recovered from the peak of neurological decline during experimental autoimmune encephalomyelitis, exhibiting axonal regeneration and eventual remyelination in the white matter tracts. There were no immunomodulatory effects of the transplanted, genetically modified haematopoietic stem cells on immune cell lineages of recipient female mice induced with experimental autoimmune encephalomyelitis. We propose that cellular delivery of Nogo receptor(1-310)-Fc fusion protein through genetically modified haematopoietic stem cells can modulate multifocal experimental autoimmune encephalomyelitis lesions and potentiate neurological recovery.

## Teaser

Nogo receptor 1 can bind myelin-associated inhibitory factors that constitute part of the extracellular milieu during experimental autoimmune encephalomyelitis to signal axonal degeneration. Ye *et al*. report that genetically modified transplantable bone marrow stem cells can deliver the Nogo receptor-Fc therapeutic protein and promote neurorepair during experimental autoimmune encephalomyelitis.

## Graphical Abstract

**Graphical Abstract:**
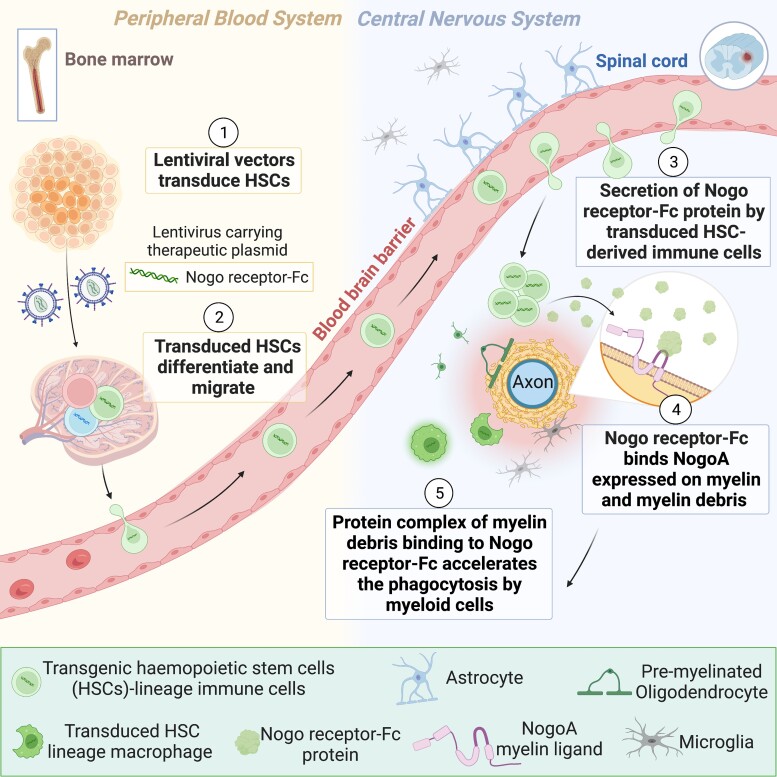

## Introduction

Multiple sclerosis is an immune-mediated neurodegenerative disorder resulting in demyelinated lesions in the brain and spinal cord, with axonal degeneration as the arbiter of permanent neurological disability.^1^ Currently, the immunomodulatory and immunosuppressive drugs available to treat multiple sclerosis may only slow disease progression, but the eventual burden of disease leads to profound neurodegeneration. As a novel means of modulating autoimmune-mediated inflammation and neurological disability, haematopoietic stem cell transplantation (HSCT) is currently being trialed to treat aggressive refractory relapsing–remitting multiple sclerosis. However, the possibility for further progression following HSCT exists in this population of treated individuals, with ∼17% of patients reported to have progressed 8 years post-transplantation (for review, see Bakhuraysah *et al*.^2^). A novel approach by which the eventual pathological sequelae and progression could be abrogated may be through limiting the deleterious accumulation of extracellular inhibitory milieu to promote neural repair, subsequent to expanding neuroinflammatory lesions.

Nogo receptor 1 (NgR1) is a high affinity receptor that binds to myelin-associated inhibitory factors (MAIFs).^3^ MAIFs can include NogoA, myelin-associated glycoprotein (MAG) and oligodendrocyte myelin glycoprotein (OMgp), which are expressed on the myelin membrane lamellae as well as within mature oligodendrocytes,^3-5^ have been shown experimentally to block neurite outgrowth.^6,7^ It has been shown that NogoA can potentiate axonal degeneration in the optic nerves and spinal cords of mice induced to develop experimental autoimmune encephalomyelitis (EAE), an animal model of multiple sclerosis.^8^

NogoA has been an attractive target in neurological research, and recently, a clinical phase I safety, tolerability and pharmacokinetics trial for relapsing–remitting multiple sclerosis has been completed (NCT01435993). This study utilized ozanezumab, a therapeutic monoclonal antibody targeting the N-terminus of NogoA, was abruptly terminated due to questions surrounding preclinical model data exclusion.^9^ A phase II study has also been completed with ozanezumab for amyotrophic lateral sclerosis, reporting no efficacy in a double-blind controlled trial.^10^ Despite termination of these previous trials due to fatal data reporting errors,^9,10^ targeting myelin ligands could be effective in treatment of specific neurological conditions with an appropriate preclinical design and therapeutic window. Indeed, the anti-Lingo 1 (NgR1 coreceptor) humanized monoclonal antibody, opicinumab, has been identified to have a clear mechanism of action in robust preclinical and clinical studies.^11-13^ Data from the RENEW trial (NCT01721161) with enrolled patients intravenously infused with opicinumab showed beneficial effects utilizing sensitive multifocal field visual evoked potential measures to assess optic neuritis latencies.^14,15^ Moreover, a phase II SYNERGY clinical trial (NCT01864148) confirmed disability improvement in 65% of relapsing–remitting multiple sclerosis subjects during the administration of opicinumab at a dosage of 30 mg/kg.^16^ However, a dose–response was observed with disability improvement being attenuated over 72 weeks, suggesting that the initial remyelination potentially being promoted by opicinumab treatment was either lost or dysfunctional with further ensuing inflammation.^16^ This has led the sponsor (Biogen Idec) to launch a Phase IIb AFFINITY trial (NCT03222973),^17^ incorporating the novel anti-Lingo 1 antibody Li81 (opicinumab)^18^ with the recruitment of the secondary Lingo 1 binding site to enhance remyelination.

It is plausible to suggest that developing biologicals such as ozanezumab or opicinumab to purely antagonize the receptors and ligands expressed upon oligodendrocyte precursor or neuronal cells may be insufficient in a disease environment that may also require active clearance of cellular debris that is inhibitory to neuroregeneration in the CNS.^19^ At the paranodal regions of myelinated nerve fibres, resident microglia or myeloid macrophages play the role in attacking myelin sheath and stripping away compact MAIF-enriched membranes during active inflammation.^19-21^ Also, subsequent deposition of extracellular myelin debris is engulfed by phagocytic microglia/macrophages which may be manipulated to actively promote CNS repair in the context of inflammation.^19-21^ Studies using the ectodomain of NgR1 demonstrate that blocking the binding of extracellular MAIFs can enhance neurological recovery following CNS injury through enhanced axonal regeneration in animal models of spinal cord injury and glaucoma.^22-26^ We postulated that functional inhibition of MAIFs in EAE by means of administrating the NgR(310)ecto-Fc decoy protein ectodomain of NgR capable of binding to MAIFs fused with immunoglobulin G (IgG)-Fc may contribute to repair following demyelination and axonopathy in the context of EAE-induced neuroinflammation.

Due to the tightly regulated blood–brain barrier (BBB) in multiple sclerosis patients, it is challenging for molecules to enter the CNS compartment. However, as multiple sclerosis and EAE exhibit large numbers of inflammatory cell infiltrates within CNS lesions, we took advantage of the properties of activated immune cells during the pathogenesis of EAE in our study. By directly genetically modifying transplantable HSCs that can differentiate into any immune cell lineage, we devised a specific cellular delivery method for the therapeutic NgR(310)ecto-Fc fusion protein, localized specifically to sites of CNS inflammatory demyelination. Delivery of the NgR(310)ecto-Fc decoy fusion protein to the inflammatory demyelinating lesion during EAE progression achieved a novel means of overcoming axonal degeneration and enhancing neurorepair. The animals transplanted with therapeutic NgR(310)ecto-Fc-overexpressing HSCs were rescued from symptoms associated with EAE. These results suggest that HSCs can be utilized as carriers of the therapeutic protein for specific targeting of EAE lesions, thereby promoting neurological recovery.

## Material and methods

### Animals

In accordance with the ARRIVE (Animal Research: Reporting of In Vivo Experiments) 2.0 updated guidelines,^27^ this original study was designed to compare female recipient C57Bl/6 mice, transplanted with littermates male donor mice HSCs either non-transduced or transduced with either empty or NgR(310)ecto-Fc vectors. Randomization occurred through allocating irradiated mice into cages, which included four HSC recipient mice per cage where a minimum of eight recipient mice were included in the transplantation experiment. These experiments were performed five times over the period of 6 years and adhered to all ethical standards to ensure mice were immediately humanely killed when they exceeded an EAE clinical score of 3 or defined animal distress using the mouse grimace scale. Age- (6–8 weeks old) male donor and age- (8–12 weeks old) female recipient C57Bl/6 wildtype mice were bred and maintained at the Alfred Medical Research and Education Precinct animal facility. The Animal Ethics Committee (AEC# E/1532/2015/M) approved the use of these mice for experimentation, in accordance with the principles of the National Health and Medical Research Council of Australia.

### Lentiviral vector design and plasmid propagation

We cloned the NgR1(1-310) ectodomain construct fused with an additional myc-tag and Fc-binding ligand into the lentiviral packaging plasmid, pLVX-EF1-IRES-ZsGreen1 (Clontech). Briefly, the NgR(310)ecto cDNA was cloned from the adult mouse brain Marathon-Ready cDNA (Clontech) and inserted into pFUSE-mIgG1-Fc plasmid (InvivoGen) to add the Fc component. The NgR(310)ecto-Fc portion was amplified and inserted into the pCMV-Tag 5 plasmid (Agilent Technologies) to add a myc-tag for tracking purposes. The total NgR(310)ecto-myc-Fc cDNA was confirmed by DNA sequencing before being ligated into pLVX-EF1α-IRES-ZsGreen1 with primers 5′-TATTTCCGGTGAATTCTTAATGAAGAGGGCG TCCT-3′ (forward) and 5′-TAGTCTCGAGGAATTCGCCCTACAGATCCTCTTC-3′ (reverse). This yielded pLVX-EF1α-NgR(310)ecto-myc-Fc-IRES-ZsGreen plasmids, were propagated by bacterial culture and purified by maxiprep kit (Qiagen).

### Lentiviral transduction into haematopoietic stem cells

Bone marrow derived lineage-negative cells extracted from male donor wildtype mice were either non-transduced or transduced with either lentivirus-carrying empty or NgR(310)ecto-myc-Fc vectors, at a multiplicity of infection (MOI) of 50. StemSpan^TM^ Serum-Free Medium (StemCell Technologies) contained L-glutamine with penicillin, streptomycin (1 × 10^5^ units/mL, Life Technologies), IL-3 (5 ng/mL), IL-6 (1 ng/mL), SCF (50 ng/mL) and Flt3 ligand (50 ng/mL; PeproTech), as HSC medium in our experiments. The vector was added to 2 mL of HSC medium in 35 mm dishes coated with 20 μg of retronectin (Clontech) and spin-infected at 1200×g at 32°C for 90 minutes. Post-centrifugation, the supernatant was removed. 1.5 × 10^6^ lineage-negative cells were seeded in 2 mL of HSC medium and spin-transduced for further 60 minutes at 32°C, 290×g. Subsequently, another 1 mL of HSC medium was added, and the cells were allowed to incubate for 72 hours.

### Irradiation and transplantation via intravenous injection

Female recipient C57Bl/6 mice were transplanted with prepared HSCs, either non-transduced or transduced with empty or NgR(310)ecto-Fc vectors. Prior to transplantation, the mice were irradiated with two separate doses of 550 cGy caesium source gamma radiation to ablate their native endogenous bone marrow. Following a 3-hours period of recovery, the mice were then intravenously injected with 1 × 10^6^-lentivirus-transduced HSCs in 200 μL mouse tonicity phosphate buffered saline (PBS) containing 2% v/v foetal bovine serum (FBS). After transplantation, all mice were monitored daily. They were weighed and humanely killed if more than 15% of their body mass had been lost overnight. They were provided with mash, jelly and 10 mM neomycin containing acidified water (pH 2.9) and kept on a heat pad until engraftment was achieved.

### Immunization with myelin oligodendrocyte glycoprotein (MOG)_35–55_

After 8 weeks of recovery post-transplantation, recipient mice underwent EAE induction by immunizing with 200 μg of encephalitogenic peptide of myelin oligodendrocyte glycoprotein (MOG_35–55_) emulsified in complete Freund’s adjuvant (CFA; Sigma-Aldrich) and supplemented with 4 mg/mL inactivated mycobacterium tuberculosis (BD Biosciences), subcutaneously injected at both sides of the lower flank. Subsequently, 350 ng pertussis toxin (List Biological Laboratories) was administered intraperitoneally, and then a further 350 ng of pertussis toxin was administered 48 hours post-induction. Overall, there were five experimental groups of mice included: (i) naïve mice (abbreviated as ‘Naïve’); (ii) non-irradiated and non-transplanted control mice with EAE induction (abbreviated as ‘EAE’); (iii) irradiated but transplanted with non-transduced HSCs control mice with EAE induction (abbreviated as ‘Mock HSCT EAE’); (iv) empty vector-transduced (abbreviated as ‘empty vector HSCT EAE’); and (v) NgR(310)ecto-Fc-transduced HSC-transplanted mice with EAE induction [abbreviated as ‘NgR(310)ecto-Fc HSCT EAE’].

### Clinical score assessment

All experimental mice were coded by the principal investigator (PI) according to the year that transplantation occurred and the treatment performed on the individual mouse number. The clinical/behavioural observations that were performed by the scientific staff and students were blinded to all treatments following randomization of mice according to cage numbers and treatment regimes. These randomized mouse categories were stored digitally by the PI on two separate devices protected by a password code. Unblinding occurred following completion of all datasets generated on excel spreadsheets. Daily clinical manifestations of immunized mice were observed and evaluated according to the previous standardized protocol during 30 days post-EAE induction.^8^ Paralysis of the mice’ limbs, legs and tail is considered as the primary criterion in this clinical score scale. The peak clinical score was assessed as 3 in our experiments (Supplementary Table 1). In accordance with ethical considerations, mice were humanely killed when their clinical scores were assessed as 4 or 5. Survival rate, mortality and disease incidence of all MOG_35–55_-induced EAE mice were also evaluated (Supplementary Table 1). A minimum of 12 mice were included in the transplantation experiments with a minimum of 8 mice included in power analysis calculations for an alpha value of <0.05 to reject the null hypothesis when comparing EAE clinical score outcomes.

### Immunoprecipitation (IP)

#### Preparation of single cell suspension

The snap-frozen spleen and spinal cords of experimental mice were dissected, fragmented and preserved in Earle’s balanced salt solution (EBSS, Life Technologies). Spleen samples were then homogenized in 1× radioimmunoprecipitation assay (RIPA) buffer (Cell Signaling Technologies) with protease (Qiagen) and phosphatase inhibitors (Calbiochem). The resulting homogenate was then centrifuged at 13 000 rpm for 20 minutes, where the protein concentrations of the supernatant collected were determined using the bicinchoninic acid protein (BCA) assay kit (Thermo Scientific).

Spinal cord fragments were processed to the single cell suspension. Tissue fragments were digested by papain dissociation kit (Worthington Biochemical Corp) at 37°C for 30 minutes and then washed with EBSS. These dissociated tissues were then triturated in ovomucoid solution (trypsin inhibitor, Sigma-Aldrich) with 0.005% w/v DNase (Roche) and filtered to obtain the homogenous suspension.

#### Immunopanning of CD11b+ macrophages

Panning dishes with 2 mL of Tris buffer (50 mM, pH 9.5) with secondary anti-rabbit IgG (Supplementary Table 2) were incubated at 4°C overnight. The next day, panning dishes coated with secondary antibody were incubated with primary antibody rabbit anti-CD11b (Supplementary Table 2) which were mainly probed against macrophages at room temperature for 2 hours. The prepared spinal cord homogeneous suspension was then panned on the pre-incubated petri dishes coated with the CD11b antibody–antigen complex, with 2 hours incubation in a 10% CO_2_ and 37°C incubator. Following incubation, CD11b+ macrophages from spinal cord single cell suspension were bound to the panning plate. The adherent cells were detached by adding 1 mL trypsin solution (0.25% v/v Trypsin EDTA, Life Technologies) with Ca^2+^ Mg^2+^ free EBSS. Next, 10% v/v FBS solution in Dulbecco's Modified Eagle Medium (DMEM) was treated in the panning plate to neutralize the trypsin and 2 mL DMEM with PBS and added to wash the panning plate, following another round of cell collection. The CD11b+ macrophage cluster was dislodged by the gentle aspiration and then harvested by centrifugation at 220×g for 15 minutes.

Purified CD11b+ macrophage clusters were then lysed in the cell lysis cocktail including 1× RIPA buffer (Cell Signaling Technology), PhosSTOP™ phosphatase inhibitor cocktail and protease inhibitor cocktail (Calbiochem). Subsequently, they were centrifuged at 13 000 rpm for 20 minutes. The supernatant was then collected and preserved at −20°C. Concentrated CD11b+ macrophage lysates containing protein of interest were assessed by BCA assay and analyzed at wavelength 562 nm to determine the lysate’s concentration.

#### IP

100 μg of protein sample was incubated overnight at 4°C with 1 μg of mouse anti-myc (Supplementary Table 2) capture antibody with constant agitation. These samples were then incubated with mouse IgG for 2 hours at room temperature. In the follow-up, 100 μL of Protein G magnetic beads (Millipore) were incubated for 30 minutes to capture the antibody–antigen complex. After completion of the incubation, samples were placed in the magnetic beads rack. Beads were magnetized and precipitated to the sides of tubes. Clearly separated supernatant was then discarded and washed in Tris-based saline with 0.1% v/v Tween-20 (TBST). 5 μL Antioxidant (Invitrogen) and 5 μL 4× LDS (lithium dodecyl sulphate) sample buffer (Invitrogen) z samples were boiled for 5 minutes at 95°C to denature proteins completely.

#### Western blot

For each western immunoblot, 5 μg protein lysates were loaded and run on 4–12% Bis-Tris gel (Invitrogen). For immunoblot analysis, protein-loaded gels were electrophoretically transferred onto polyvinylidene fluoride (PVDF) membranes (Millipore). The membranes were blocked in 5% skim milk in TBST (blocking buffer) and primary antibodies (Supplementary Table 2) diluted in blocking buffer were added and incubated overnight at 4°C. After thoroughly washing the membranes with TBST, secondary horseradish peroxidase (HRP)-conjugated antibodies (Supplementary Table 2) were added and incubated for 2 hours at room temperature. Immunoreactive proteins were detected using the enhanced chemiluminescence (ECL) prime chemiluminescence kit (GE healthcare).

#### Immunofluorescence

All mice were humanely killed with CO_2_ inhalation and perfused with 1× mouse tonicity PBS followed by 4% paraformaldehyde (PFA). The spinal cords were dissected and post-fixed in 4% PFA for 24 hours, at 4°C. Subsequently, samples were then cryo-protected in 15% and 30% w/v sucrose for 24 hours each at 4°C. Samples were then embedded in OCT (optimal cutting temperature) compound (Tissue-Tek) and frozen in isopropanol cooled on dry ice. Longitudinal sections were cut at 10 μm on a cryostat (Leica Microsystems) and mounted on microscope slides. Sections were then blocked and permeabilized in 10% v/v normal goat serum and 0.3% v/v Triton X-100 in PBS for 1 hour at room temperature. Primary antibodies (Supplementary Table 2) diluted in the blocking buffer (either 5% v/v normal goat serum or normal donkey serum and 0.1% v/v Triton X-100) were applied on tissue sections overnight at 4°C. Secondary antibodies (Supplementary Table 2) diluted in the blocking buffer were applied on tissue sections for 2 hours at room temperature. Sections were counterstained with DAPI (Invitrogen) for 15 minutes at room temperature and then mounted with fluorescent mounting medium (Dako).

#### Imaging analysis

Images were taken using a Nikon A1R inverted confocal microscope with 20× glycerol objective. The captured multichannel images were separated into individual channels by ImageJ (Fiji), measuring the number of cells of interest, intensity density of the cell marker fluorescence and intensity density of myelin debris. The intensity profiling of protein of interest was measured as integrated density exclusively in ZsGreen-positive areas and normalized by ZsGreen-negative areas in ImageJ (Fiji). Inflammatory and peri-plaque regions within white matter were manually defined using customized tools in Fiji software as the region of interest (ROI). ROI was traced in accordance with the DAPI nuclei for all immunofluorescence analysis. The region of aggregated DAPI-labelled nuclei and ZsGreen expression is referred to as the inflammatory region (IR), while evenly dispersed DAPI nuclei is defined as the peri-plaque white matter (PPWM). All immune cell profiles and histopathological outcomes in transplanted C57Bl/6 female recipient mice with MOG_35–55_-induction were assessed by observers in a blinded manner using codes kept by the PI. Decoding occurred following the compilation of the dataset.

### Histopathological staining

The perfusion-fixed spinal cords were prepared embedded in resin as described below for transmission electron microscopy. Spinal cord sections were stained using luxol fast blue (LFB), periodic acid Schiff (PAS) and Bielschowsky silver stains as previously described.^28^ Images were captured under a brightfield Olympus BX51 microscope.

#### Analysis of dystrophic axons by toluidine blue-stained semithin sections

1 µm semithin sections were cut using a standard microtome. Sections were incubated with 1% w/v toluidine blue in 1% w/v sodium borate at 60°C for 30 seconds, then washed in running dH_2_O. Subsequently sections were mounted in DPX mounting medium. Images were taken using an Olympus BX51 brightfield microscope with ×100 oil objective.

### Immunogold-labelled electron microscopy (EM)

Spinal cords were fixed overnight at 4°C with 0.1 M phosphate buffered 2% w/v EM-graded PFA and 0.2% w/v glutaraldehyde. The fixed samples were embedded in 12% w/v gelatin in 0.1 M phosphate buffer at 37°C and allowed to set at 4°C before being cut into small cubes measuring ∼0.5 mm on each edge. The gelatin embedded tissues were infiltrated with 2.3 M sucrose in 0.1 M phosphate buffer at 4°C overnight on a rocker. The sucrose infiltrated gelatin blocks were mounted on cryo-pins and then frozen in liquid nitrogen for cryo-ultramicrotomy. Frozen samples were trimmed at −100°C and sectioned at −120°C using a Cryo-EM UC7 ultramicrotome (Leica) equipped with a 45° diamond cryo-trimming knife (Diatome) and a 35° diamond cryo-immuno knife (Diatome). Cryo-sections were retrieved by pick-up loop with a droplet of phosphate buffered 1% w/v methyl cellulose and 1.15 M sucrose and then deposited on carbon-coated formvar grids for immunolabelling. The grids were prepared for immunolabelling by melting up-side-down on PBS at 37°C and then rinsing the grids on droplets of 0.02 M glycine in PBS. The grids were blocked with 1% w/v bovine serum albumin (BSA) in PBS, incubated with anti-myc-tag (Supplementary Table 2) in 1% w/v BSA in PBS at room temperature for 1 hour and incubated with a bridging rabbit anti-biotin (100–4198, 1:10,000, Rockland) and then rinsed with 0.1% w/v BSA in PBS. The grids were then incubated with Protein-A conjugated 10 nm gold particles diluted (1:50, Cellbiology, Utrecht) in 1% w/v BSA in PBS for 20 minutes at room temperature, rinsed with PBS. After stabilization of the reaction by 1% w/v glutaraldehyde in PBS for 5 minutes, grids were rinsed in distilled water. Finally, the grids were stained with 2% w/v uranyloxaalacetate (pH 7.0) for 5 minutes at room temperature and 0.4% w/v uranyl acetate in 1.8% w/v aqueous methyl cellulose (pH 4) for 5 minutes at 4°C and dried in a thin film of the final stain in the centre of a wire loop. EM imaging was done on JEM-1400Flash (Jeol).

### Transmission electron microscopy (TEM)

Spinal cords were fixed in 2% w/v paraformaldehyde and 2.5% w/v glutaraldehyde in 0.1 M sodium cacodylate (pH 7.4) supplemented with 5 mM calcium chloride and 10 mM magnesium chloride for 2 hours at room temperature. They were then washed and post-fixed in 1% w/v osmium tetroxide and 1.5% w/v potassium ferricyanide in 0.065 M sodium cacodylate (pH 7.4). Cells were then scraped, pelleted and processed by gradual dehydration in ethanol (70%, 80%, 90%, 95% and 100%) and propylene oxide (100%). This was followed by embedding in epon resin and then left to polymerize for 48 hours at 60°C. Ultrathin (80 nm) sections were cut by a Leica Ultracut UCT and imaged on Jeol 1400PLUS. The diameter of the inner and outer ring of the axons was measured on ImageJ, and the *g* ratio was calculated using the following formula.

$$g\;\mathrm{ratio}=\;\frac{\mathrm{Inner}\;\mathrm{ring}\;\mathrm{axon}\;\mathrm{diameter}}{\mathrm{The}\;\mathrm{total}\;\mathrm{outer}\;\mathrm{ring}\;\mathrm{axon}\;\mathrm{diameter}}$$

### *Ex vivo* MRI

The L1 to L5 spinal cord segments were dissected out for imaging due to the ascending paralysis in the EAE model with the peak of disease (EAE clinical score 3) exhibiting numerous inflammatory demyelinating lesions. After clearing excess paraformaldehyde from the spinal cord segments with PBS at 4°C, they were then transferred and immersed into a fomblin-filled tube. The spinal cord samples were examined by diffusion tensor imaging (DTI) using a 9.4 Tesla Bruker MRI scanner at the Monash Biomedical Imaging facility at the Alfred Research Alliance precinct. DTI parameters included the following; repetition time/echo time, 1000/29 ms; matrix, 192 × 144 × 64; field of view, 15.36 × 11.52 × 5.44 mm^3^, and *B*-value of 4000 s/mm^2^. Structural and microstructural analyses of spinal cord DTI data were performed. Firstly, the white matter (WM) and grey matter (GM) were segmented using ITK-SNAP software, followed by calculation of the volume ratio of GM versus WM. Microstructural analysis of the spinal cord L1–L5 was performed by assessing axial diffusivity (AD) in three ROIs, the dorsal tract, the dorsal–lateral WM and the ventral WM. AD images were reconstructed from denoised DTI images using MRtrix3 software. Acquisition and analysis of MRI data were performed blindly.

### Single molecule array (SIMOA)

Neurofilament light chain (NfL) levels in the serum of mice were assessed using SIMOA HD-X Analyser^TM^ (Quanterix, Billerica, MA, USA). The blood samples were collected by submandibular bleeding from all experimental mouse groups at the onset and progressive stage of EAE and were stored at −20°C freezer. The blood samples were centrifuged at 10 000 rpm for 20 minutes at 4°C to extract the serum. 42 μL of serum from each sample was isolated to perform immunoassays in duplicate using the Simoa® NF-light^TM^ Advantage Kits as per the manufacturer’s instructions, with the exception that samples were run using an 8× benchtop dilution rather than a 4× machine dilution. Experimenters conducted the immunoassays under a blinded condition. All samples measured above the lower limit of quantification (0.174 pg/mL) were within the assay dynamic range (1800 pg/mL).

### DigiGait^TM^ animal behaviour analysis

The gait performance was monitored by the DigiGait^TM^ imaging system and evaluated by the DigiGait^TM^ 15.0 analysis software (Mouse Specifics).^28^ Mice paw prints were captured by a transparent plastic treadmill and a high-speed digital camera. The tails of the mice were dyed black to enhance the contrast for the automated analysis. The mice were initially habituated to the treadmill before the day of testing where the training data were excluded from the final analysis. To measure the maximum speed of the mice, the speed of the treadmill was incrementally increased by 5 cm/s from 0 cm/s to 15 cm/s, at a 5 s interval (0, 5, 10 and 15 cm/s), where a video sequence of 5 seconds duration with the maximum speed was recorded.

### Statistics

Data were analyzed using GraphPad Prism 9.2 software. The normality of data distribution was validated by Shapiro–Wilk test and Kolmogorov–Smirnov test. Unpaired two-tailed *t*-test was performed for the normally distributed dataset. Welch’s correction was performed for dataset with unequal variances. Mann–Whitney two-tailed U test was applied in the non-normally distributed dataset. Ordinary one-way ANOVA with Tukey’s *post hoc* test was applied for comparing the statistical outcome of one variable in multiple groups that pass the assumption of homogeneity of variance (Brown–Forsythe test, *P* > 0.05). Otherwise, Kruskal–Wallis one-way analysis with Dunn’s *post hoc* test was used for analyzing multiple groups with the non-normal distribution. Two-way ANOVA mixed-effect model with Tukey’s *post hoc* test was used for analyzing two variables in multiple groups. Greenhouse–Geisser correction was used for adjusting the violated sphericity. All statistical analyses and *n* numbers were specified within the figure legends. Data were expressed as mean ± standard error of the mean (SEM), *P*-values of <0.05 were considered as significant unless otherwise specified. Linear regression model was used in *g* ratio analysis. *R*^2^, slope and intercept were shown on figures.

### Data availability

The authors confirm that the data supporting the findings of this study are available within the article and its Supplementary material. Derived data supporting the findings of this study are available from the corresponding author on request.

## Results

### NgR(310)ecto-Fc-transduced HSC recipient animals clinically recovered from the peak of disease

**Figure 1:**
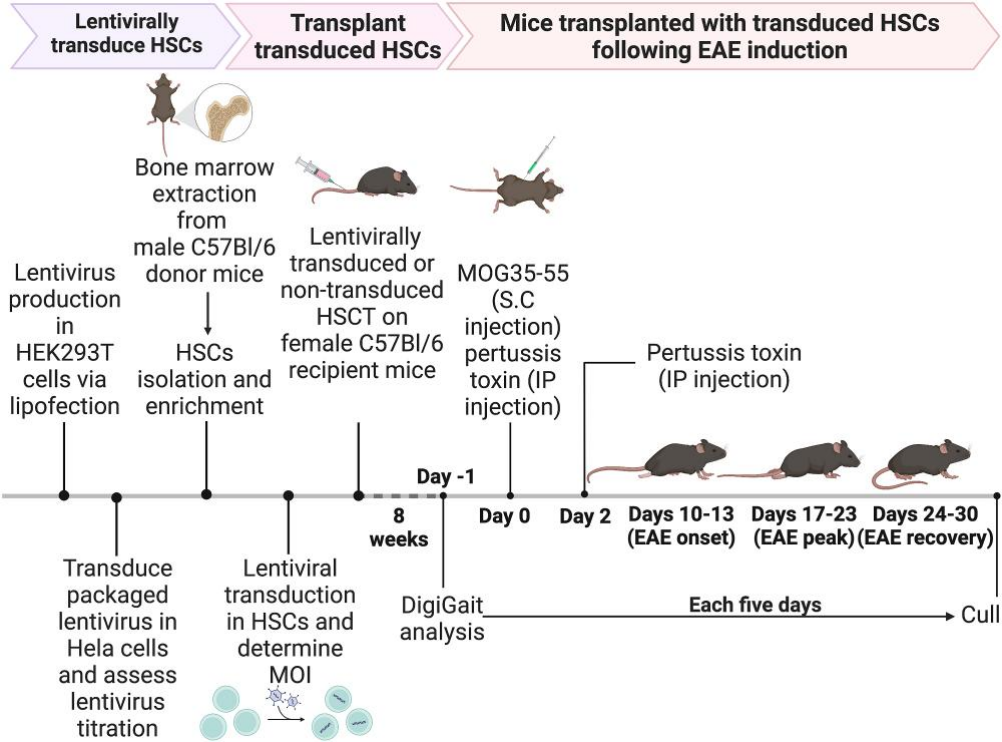
**Schematic illustration of the *in vitro* and *in vivo* experimental timeline.** Lentivirus is first packaged in HEK 293T cells and lentivirus titres achieved after infection of Hela cells in culture. HSCs isolated from C57BL6 male donor mice are transduced by packaged lentivirus. The transduced HSCs are then transplanted into female recipient littermates. After 8 weeks of recovery following engraftment, mice transplanted with transduced HSCs are induced with EAE by MOG immunization. Once EAE is induced in animals, the disease course is followed for up to 30 days.

**Figure 2:**
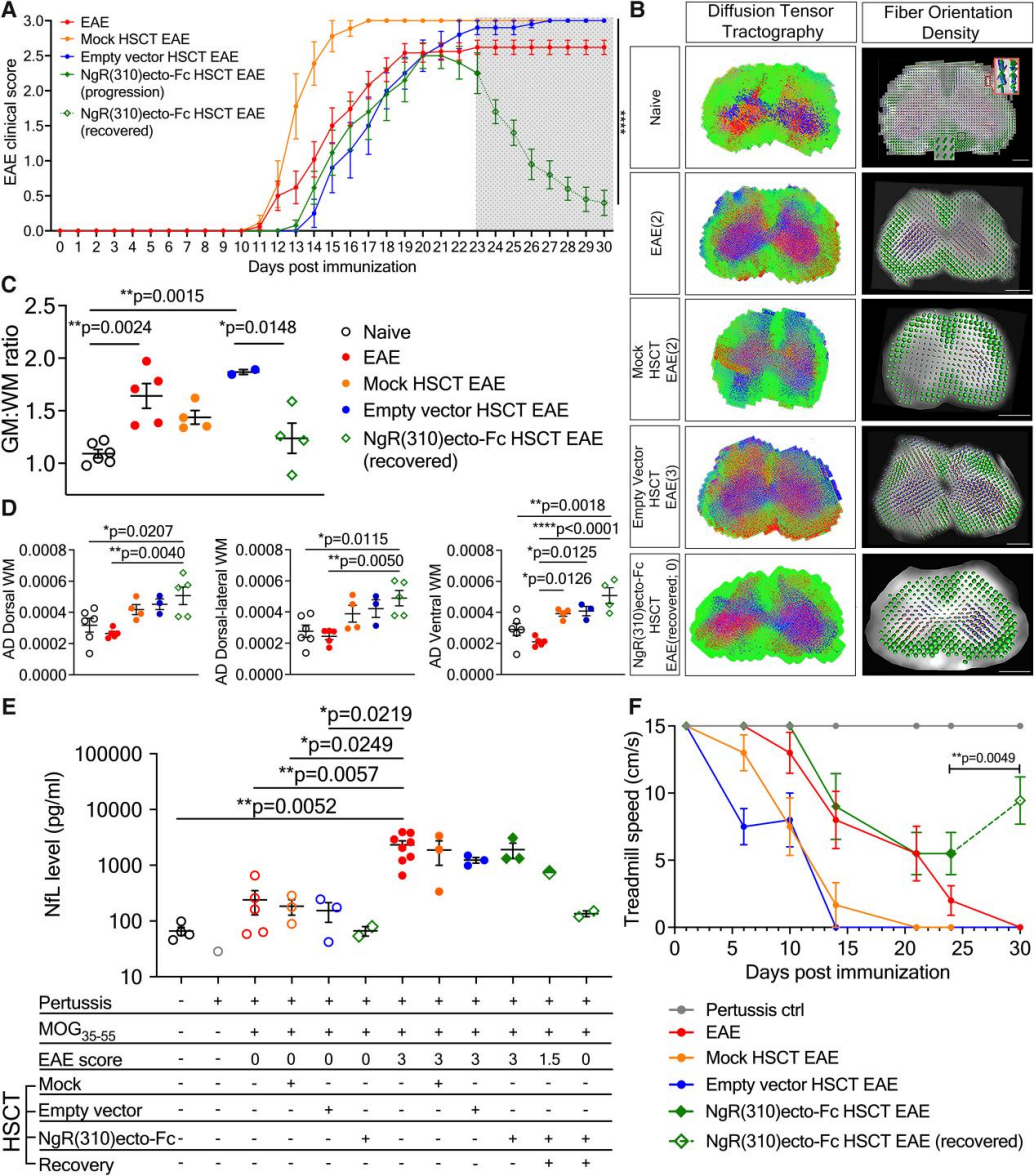
**Development of lentiviral vectors and multifaceted recovery outcome of EAE clinical score in NgR(310)ecto-Fc-transduced HSC recipient mice.** (**A**) Following MOG_33–55_-induced EAE, NgR(310)ecto-Fc HSCT recipient mice at Day 30 (*n* = 13, each data point plotted on the graph represents the mean value of EAE score) recovered from the peak stage of EAE (clinical scores 3) when compared to EAE (*n* = 25), mock HSCT EAE (*n* = 9) and empty vector HSCT EAE (*n* = 10) groups. Data are represented as mean ± SEM. Two-way ANOVA mixed-effect model with Tukey’s *post hoc* test. ****P < 0.0001. (**B**) Tractography and fibre orientation density were colour coded. Tractography indicated the diffusion of lateral fibres (blue), anteroposterior fibres (red) and rostral-caudal fibres (green) in the lumbar spinal cord sections, while fibre orientation density estimated the orientation of a single fibre shown as a voxel with gradients according to the *x* (red), *y* (green) and *z* (blue) directions. The single voxel is presented in a red box. Mice with higher EAE clinical scores displayed striking disarray in their fibre tracts with sites of lesions in red on the tractography images. Scale bar = 500 µm. (**C**–**D**) The volume ratio of grey matter versus white matter (**C**) in clinically recovered NgR(310)ecto-Fc HSCT recipients showed a closer ratio to the basal level with a higher WM AD (**D**) in regions of interest compared to empty vector HSCT recipients. *n* = 3–6. One-way ANOVA and Tukey’s *post hoc* test. *P < 0.05, **P < 0.001, ****P < 0.0001. (**E**) SIMOA detected decreased serum NfL in NgR(310)ecto-Fc HSCT recipients with an EAE clinical score of 0 compared with empty vector HSCT recipients with an EAE clinical score of 3. *n* = 3–5, except for the mouse group injected with pertussis toxin (*n* = 1) and the mouse group transplanted with NgR(310)ecto-Fc HSCs prior to EAE induction (*n* = 2). One-way ANOVA with Tukey’s *post hoc* test. *P < 0.05. (**F**) DigiGait^TM^ analysis demonstrated decreased treadmill speed in all experimental groups from post-EAE induction Day 10, but the speed of the clinically recovered NgR(310)ecto-Fc-transduced group increased at Day 30. The treadmill of the NgR(310)ecto-Fc-transduced group was significantly higher than that of the empty vector-transduced group at Day 30 (individual data points plotted on the graph represent the mean value of the treadmill speeds for each group of mice with and without transplantation). Pertussis ctrl: *n* = 3, EAE and mock HSCT EAE, empty vector HSCT EAE, NgR(310)ecto-Fc HSCT EAE: *n* = 10. Mann–Whitney two-tailed *t*-test. *P < 0.05.

Lethally irradiated mice were transplanted with lentivirally transduced HSCs carrying either empty vectors or NgR(310)ecto-myc-Fc vectors (also known as NgR(310)ecto-Fc) and immunized with MOG_35–55_ peptide to mimic immune-mediated demyelination, as illustrated in the schematic (Fig. 1). Strikingly, recipients transplanted with NgR(310)ecto-Fc-transduced HSCs, following EAE induction and fulminant neuroinflammation, exhibited significantly reduced clinical scores after the peak stage of disease symptoms, demonstrated recovery following clinical neurological decline associated with EAE (Fig. 2A). Importantly, clinical recovery in recipients transplanted with NgR(310)ecto-Fc HSCs was identified by diffusion weighted *ex vivo* DTI-MRI, whereby the fibre tract distribution was exhibited by tractography (Fig. 2B), and the integrity of axons in the neurologically recovered mice was assessed by spinal cord grey and white matter volume ratios along with axial diffusivity measurements (Fig. 2C and D; apparent diffusion coefficient (ADC), fractional anisotropy (FA) and radial diffusivity (RD) were assessed for all transplantation paradigms compared to control mice in Supplementary Fig. 8A). Initial spectral confocal reflection (SCoRe) microscopy analysis of spinal cord white matter tracts of EAE-induced HSCT recipient mice identified a significant reduction in the change in fluorescence intensity attributed to myelin debris, during the progression and recovery of EAE, in the NgR(310)ecto-Fc-transduced recipients (Supplementary Fig. 8B–D). Axonal damage was assessed by measuring the levels of NfL in serum from mouse cohorts. Randomized and double-blinded analyses confirmed the reduction in NfL levels in partially (EAE clinical score 1.5) and completely recovered (EAE clinical score 0) NgR(310)ecto-Fc-transduced HSC recipients, suggesting the potential of NfL as a molecular biomarker in our preclinical model (Fig. 2E). Analysis of locomotor performance through DigiGait^TM^ analysis demonstrated an enhanced treadmill speed during the period of recovery time in mice transplanted with NgR(310)ecto-Fc-transduced HSCs (Fig. 2F).

Secretion of the cellular NgR(310)ecto-Fc fusion protein from transfected HEK293T cell lysates was confirmed by western blotting, as indicated by the expression of full-length protein upon probing with anti-NgR1 and anti-Fc antibody (Supplementary Fig. 1B). Following transplantation in recipient female mice, we obtained sera and spleen tissue lysates that were collected at the peak of EAE from both empty- and NgR(310)ecto-Fc vector HSC transduction groups, demonstrating translation and secretion of NgR(310)ecto-Fc fusion protein from immune lineage cells (Supplementary Fig. 2C–F). Further analyses confirmed the presence of NgR(310)ecto-Fc-transduced cells within the periphery as the ZsGreen fluorescent reporter was detected by flow cytometry in the bone marrow, peripheral blood circulation and lymph node, as well as c-Kit+ haemopoietic cell population visualized in the bone marrow of recipient mice (Supplementary Fig. 3A and B). Fluorescent *in situ* hybridization (FISH) analysis within the spleen and spinal cord demonstrated definitive ZsGreen+ immune lineage cells labelled for the Y-chromosome, confirming lineage differentiation of transplanted HSCs from male mice in female recipients during the peak stage of EAE (Supplementary Fig. 3C). Importantly, the percentage of mature immune cell populations in the recipient EAE cohorts with either empty vector-, NgR(310)ecto-Fc vector-transduced HSCT or non-transduced mock HSCT was similar to EAE cohorts without transplantation, demonstrating no significant differences among mature immune lineage cells in peripheral blood and lymph node (Supplementary Fig. 4B and C). Furthermore, CD19+ (B cell) and CD3e+ (T cell) immune cell populations were observed in the spleen, along with infiltrates in inflammatory demyelinating lesions of spinal cords of NgR(310)ecto-myc-Fc vector-transduced HSCT recipient mice at the peak stage of disease (Supplementary Fig. 4D–G). These data suggest that no significant modulation could be observed in the peripheral immune profile following the lentivirus transduction of HSCs with or without NgR(310)ecto-myc-Fc after prominent lymphoid differentiation.

### Delivery of the NgR(310)ecto-Fc fusion protein to inflammatory demyelinating lesions within the CNS at the peak stage of EAE shows increased numbers of CD11b+ macrophages

**Figure 3:**
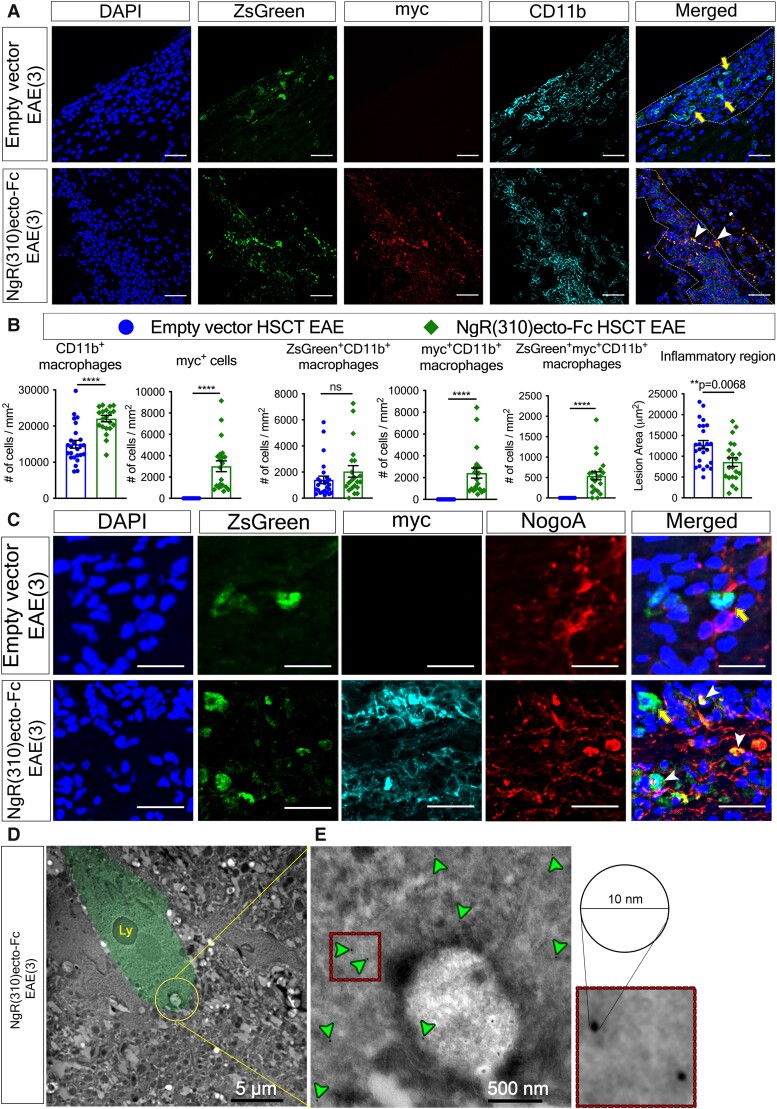
**The elevated population of CD11b+ macrophage infiltrates exhibited notable phagocytic ability within inflammatory spinal cord lesions of NgR(310)ecto-Fc-transduced HSC-transplanted recipients at the peak stage of the disease (EAE clinical score 3).** (**A**) Immunohistochemical staining of ZsGreen (green), myc (red) and CD11b (cyan) within inflammatory spinal cord lesions of the empty vector and NgR(310)ecto-Fc HSCT recipient with EAE clinical score 3. ZsGreen lineage CD11b+ macrophage infiltrates colocalized with myc (white arrowheads) or without myc (yellow arrows) are shown. Scale bar = 50 µm. (**B**) The number of CD11b+ macrophages, myc+ cells, myc+CD11b+ macrophages, ZsGreen+CD11b+ macrophages, ZsGreen+myc+CD11b+ macrophages per inflammatory lesion area (mm^2^) and area of active lesions of the spinal cord were quantified in the empty vector and NgR(310)ecto-Fc HSCT mice at the peak stage of EAE. *n* = 3 per group. Data are represented as the mean ± SEM. Mann–Whitney two-tailed *t*-test. ****P < 0.0001. Individual data points represent the analysis of cell numbers obtained from inflammatory versus non-inflammatory regions of spinal cord (longitudinal sections) of experimental mice. (**C**) Immunohistochemical imaging of ZsGreen (green), myc (cyan) and NogoA (red) within inflammatory spinal cord lesions of the empty vector and NgR(310)ecto-Fc HSCT recipient with EAE clinical score 3. ZsGreen lineage-differentiated macrophages phagocytosing NogoA (white arrowheads) or not phagocytosing NogoA (yellow arrows) were shown in the spinal cords of the empty vector- and NgR(310)ecto-Fc HSCT recipient animals with EAE clinical score 3. Scale bar = 20 µm. (**D**) Immunogold-labelled electron microscopy visualized the engulfment of myc-tagged protein complex of NgR(310)ecto-Fc-bound myelin debris into the lysosome (Ly) by macrophages. (**E**) High-magnification view of gold particles conjugated to a myc tag against NgR(310)ecto-myc-Fc fusion protein as individual black dots (with a diameter of 10 nm) within the macrophage cytoplasm or distributed surrounding the membrane of the macrophage (green arrowheads).

One of the key pathological hallmarks of multiple sclerosis and EAE is inflammatory cell infiltrates that mobilize to the CNS via extravasating through a leaky BBB.^29^ We aimed to exploit this biological property of activated immune cells and thereby directly deliver the NgR(310)ecto-Fc protein to the inflammatory demyelinating lesions within the CNS from lineage-differentiated HSCs. We detected the localization of transduced infiltrated leucocytes within inflammatory lesions of spinal cords in HSC recipient mice (Supplementary Fig. 4F and G), with CD11b+ macrophages representing the predominant population of differentiated immune cells (Fig. 3A). We identified a significant increase in the number of CD11b+ cells within NgR(310)ecto-Fc HSCT mice when compared to the empty vector-transduced mice (Fig. 3B, the first graph). However, we did not demonstrate a significant difference in the numbers of ZsGreen+ lineage-differentiated CD11b+ macrophages between the empty vector and NgR(310)ecto-Fc-transduced HSC-transplanted groups, indicating that NgR(310)ecto-Fc did not affect macrophage-monocytic lineage differentiation (Fig. 3B, the third graph). We further quantified the population of CD11b+ macrophages colocalized with either myc (Fig. 2B, the fourth graph) or ZsGreen and myc (Fig. 2B, the fifth graph) in the NgR(310)ecto-Fc vector-transduced group, which attempted to differentiate whether NgR(310)ecto-Fc protein that was phagocytosed by total peripheral macrophages or that was secreted/phagocytosed by only ZsGreen lineage macrophages in smaller and less densely packed inflammatory infiltrates within lesions. We showed an ∼5-fold increase in the absolute numbers of myc+CD11b+ cells that were myc+ (2420 ± 467.1/mm^2^) compared with ZsGreen+myc+CD11b+ cells (542.0 ± 98.60/mm^2^) in NgR(310)ecto-Fc-transduced HSC-transplanted mice, suggesting that non-transduced phagocytes participated in the engulfment of the NgR(310)ecto-Fc fusion protein (Fig. 3B). Furthermore, the engulfment of the NogoA protein within ZsGreen+ myc+ HSC-derived CD11b+ macrophages was also observed in these lesions, suggesting the potential presence of NgR(310)ecto-myc-Fc-MAIF complex (Fig. 3C). These data were further supported by immunogold-labelled myc+ particles phagocytosed in the phagosome of CD11b+ macrophages, clearly showing phagocytic uptake in NgR(310)ecto-Fc vector-transduced HSC recipient animals (Fig. 3D and E).

**Figure 4:**
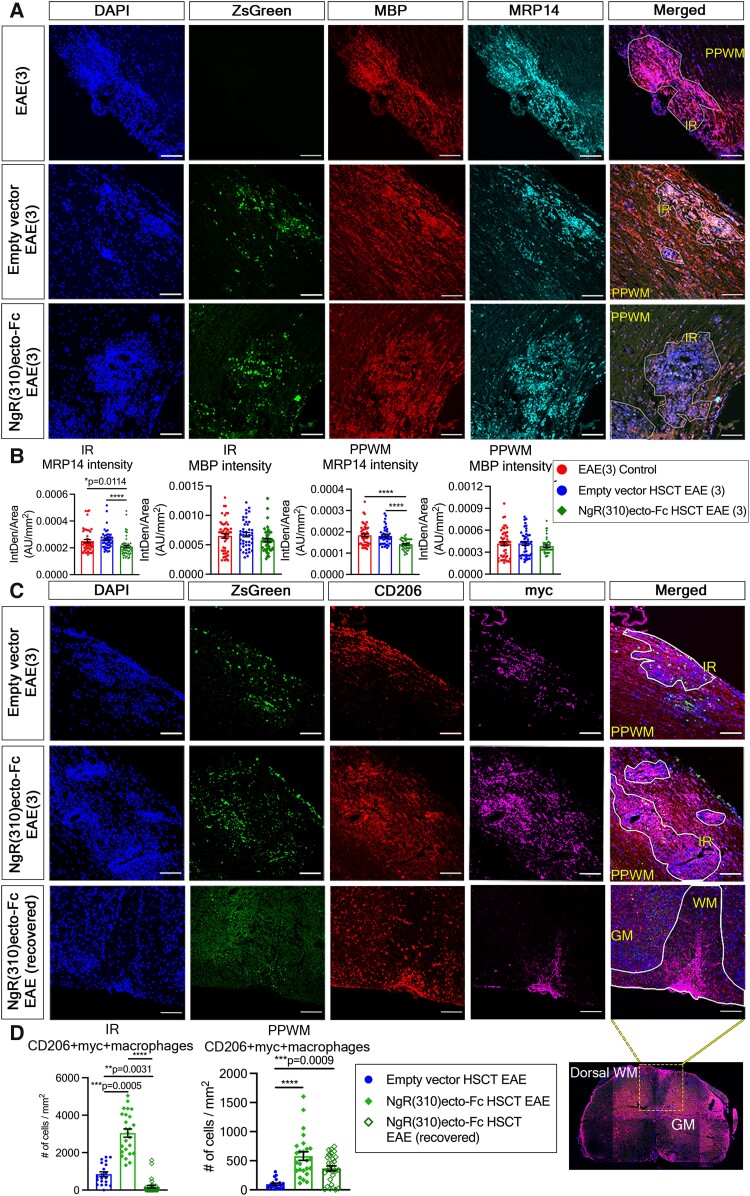
**Increased population of anti-inflammatory CD206+ macrophages with concomitant decreased pro-inflammatory MRP14+ macrophages within the inflammatory spinal cords of NgR(310)ecto-Fc vector-transduced HSC-transplant recipient animals at the peak stage of the disease (EAE clinical score 3).** (**A**) Immunohistochemical staining of ZsGreen (green), MBP (red) and MRP14 (cyan) in ZsGreen lineage macrophages within inflammatory regions (IRs) and peri-plaque white matter (PPWM) of the spinal cords of the empty vector and NgR(310)ecto-Fc HSCT recipient animals at EAE clinical score 3. Scale bar = 50 µm. (**B**) The intensity density of MRP14 fluorescence was quantified per IR and PPWM area (mm^2^) in the EAE control, empty vector and NgR(310)ecto-Fc HSCT mice at the peak stage of disease. *n* = 3 per group. Kruskal–Wallis one-way analysis with Dunn’s *post hoc* test. Data are represented as mean ± SEM. *P < 0.05, ****P < 0.0001. Individual data points represent the analysis of fluorescence intensity obtained from inflammatory versus non-inflammatory regions of spinal cord (longitudinal sections) of experimental mice. (**C**) Immunohistochemical staining of ZsGreen (green), CD206 (red) and myc (magenta) in transduced HSC-derived macrophages within IR and PPWM in the spinal cords of the empty vector- and NgR(310)ecto-Fc HSCT recipient animals at the peak stage of EAE (clinical score 3), and NgR(310)ecto-Fc HSCT recipient animals at the recovery stage. Scale bar = 100 µm. (**D**) The number of CD206+myc+ macrophages was quantified per IR and PPWM area (mm^2^) in the empty vector, NgR(310)ecto-Fc HSCT mice and clinically recovered NgR(310)ecto-Fc HSCT mice during EAE progression. *n* = 3 per group. Kruskal–Wallis one-way analysis with Dunn’s *post hoc* test. Data are represented as mean ± SEM. *P < 0.05, **P < 0.01, ****P < 0.0001. Each data point represents the analysis of cell numbers obtained from the individual longitudinal spinal cord sections from experimental mice.

Peripheral macrophages and endogenous microglia are the main effectors in multiple sclerosis and MOG_35–55_-induced EAE. Previous studies suggest that during MOG_35–55_-induced EAE, a shift towards the pro-inflammatory phenotype of classically activated macrophage/microglia occurs, promoting inflammation and disease progression.^30^ A shift towards an anti-inflammatory phenotype of alternatively activated macrophage/microglia has been associated with evidence of neurotrophic support potentiating repair and regeneration.^31-33^ Either mice administrated with the transplantation of NgR(310)ecto-Fc-transduced HSCs or the empty vector-transduced HSCs, similar demyelinating outcomes based on MBP myelin protein expression within inflammatory, and peri-plaque white matter regions were observed in these cohorts at clinical score 3 (Fig. 4A and B). In the current study, we demonstrated a decrease in the number of classically activated macrophages identified by the numbers of myeloid-related protein (MRP)-14+^34,35^ and iNOS+ myeloid cells^36,37^ in demyelinating spinal cord sections of NgR(310)ecto-Fc HSCT EAE mice at the peak stage of disease (Fig. 4A and B; Supplementary Fig. 5A and B). Thus, we further characterized the effects of NgR(310)ecto-Fc on the expression of alternatively activated macrophages (CD206+ and Arg1+)^37-40^ by analyzing infiltrates throughout spinal cord lesions at the peak stage of EAE and compared this with mice that had neurologically recovered (EAE score 0) (Fig. 4C and D; Supplementary Fig. 5C and D). Significant numbers of alternatively activated CD206+ macrophages in the spinal cords of NgR(310)ecto-Fc HSCT recipient mice were colocalized with cells expressing ZsGreen and myc, indicating that these cells were derived from the transplanted HSCs (Fig. 4C). There were ∼3-fold higher numbers of CD206+myc+ cells present within the inflammatory regions of spinal cords of NgR(310)ecto-Fc HSCT recipient mice compared to empty vector HSCT recipient mice (3044 ± 222.1/mm^2^ versus 845.8 ± 126.8/mm^2^, *P* = 0.0005) **(**Fig. 4D), suggesting that other endogenous CD206+ZsGreen− cells were potentially uptaking the NgR(310)ecto-Fc produced by transduced and transplanted HSC-derived cells with lineage differentiation at the peak stage of disease. A visible decrease in the expression of CD206+myc+ cells within the inflammatory regions were observed in clinically recovered NgR(310)ecto-Fc vector-transduced HSC recipient mice compared to mice at the peak stage of EAE (180.4 ± 73.78/mm^2^ versus 3044 ± 222.1/mm^2^, *P* < 0.0001; Fig. 4D). Similarly, in the peri-plaque regions of neuroinflammation, there was an increase in CD206+myc+ expression in NgR(310)ecto-Fc HSCT recipient mice compared to empty vector HSCT recipient mice (578.7 ± 73.95/mm^2^ versus 95.78 ± 18.99/mm^2^, *P* < 0.0001; Fig. 4D). The numbers of CD206+myc+ cells were also reduced in clinically recovered NgR(310)ecto-Fc HSCT recipient mice when we compared the same recipient groups of mice at the peak stage of EAE (366.7 ± 42.90/mm^2^ versus 578.7 ± 73.95/mm^2^, respectively; Fig. 4D). These data collectively suggest that the active production of NgR(310)ecto-Fc fusion protein within and around spinal cord neuroinflammatory lesions may shift the macrophage phenotype towards an alternate neurorepair and neuroprotective phenotype.

**Figure 5:**
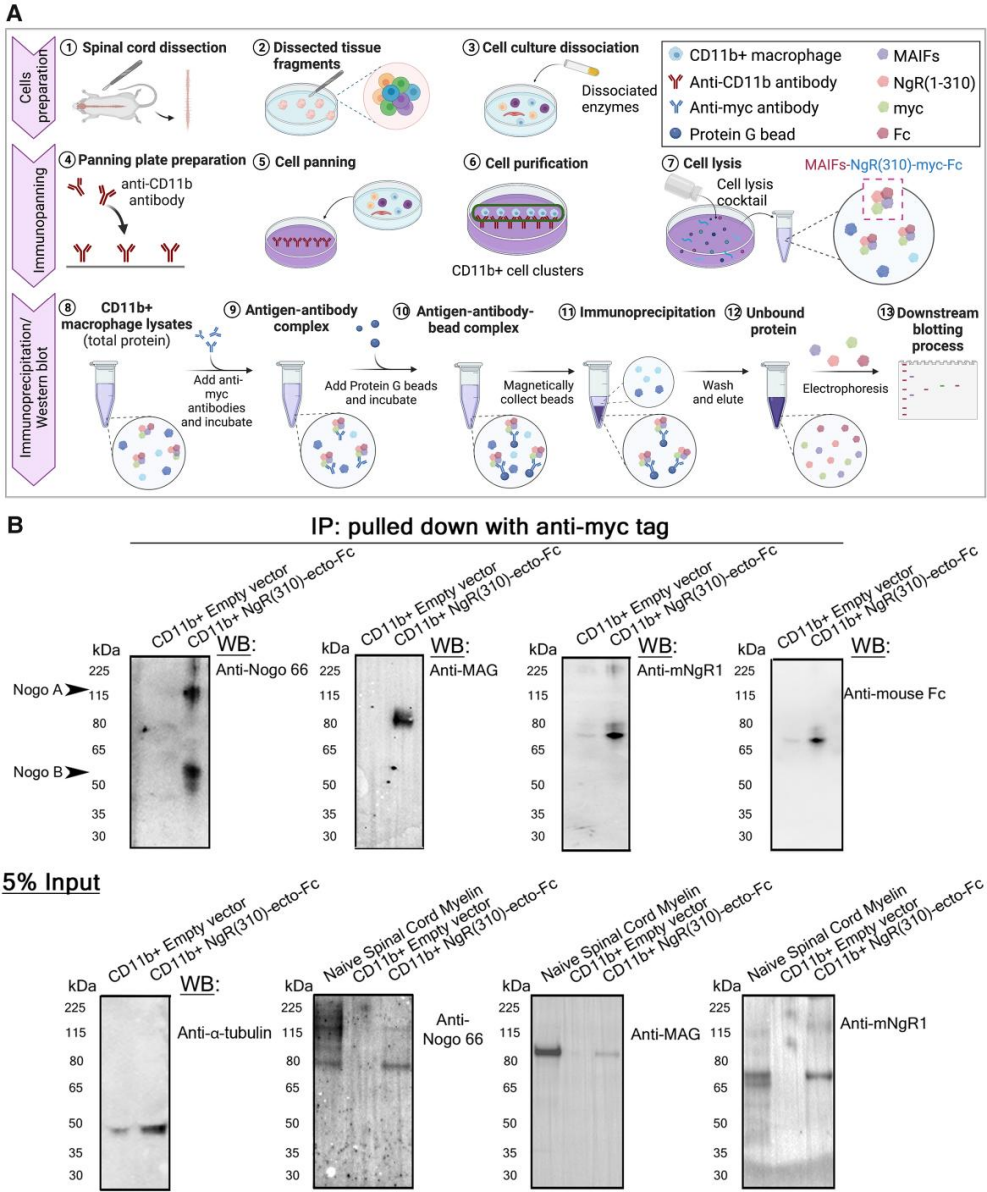
**The role of microglia and macrophages in the phagocytosis of extracellular myelin protein that were bound to interstitially produced NgR(310)ecto-Fc was validated by immunoprecipitation.** (**A**) Schematic illustration of the purification of CD11b+ macrophages by immunopanning and the pull-down of myc-tagged myelin debris by immunoprecipitation. The schematic diagram was generated by BioRender. (**B**) The expression of Nogo 66, MAG, NgR1 and Fc protein was detected in myc-tagged CD11b+ macrophage lysates isolated from the spinal cord of NgR(310)ecto-Fc HSCT mice at EAE peak stage. Direct western blotting detected 5%-input Nogo 66, MAG, NgR1 and tubulin protein in both transduced-HSCT mice. See Supplementary Fig. 9 for uncropped blots.

The evidence that large amounts of myelin proteins were being engulfed by macrophages within neuroinflammatory lesions at the peak stage of EAE in the NgR(310)ecto-Fc HSCT recipient mice was demonstrated by isolating CD11b+ cells using an immunopanning procedure of homogenized lumbosacral cord spinal cords from these mice. NgR1 and Fc protein, along with Nogo 66 and MAG which are components of MAIFs, were immunoprecipitated by anti-myc antibody in NgR(310)ecto-Fc HSCT recipients, while there was no detection from empty vector HSCT recipient mice (Fig. 5B).

### Transplanted HSCs transduced with the NgR(310)ecto-Fc vector may enhance axonal regeneration

**Figure 6:**
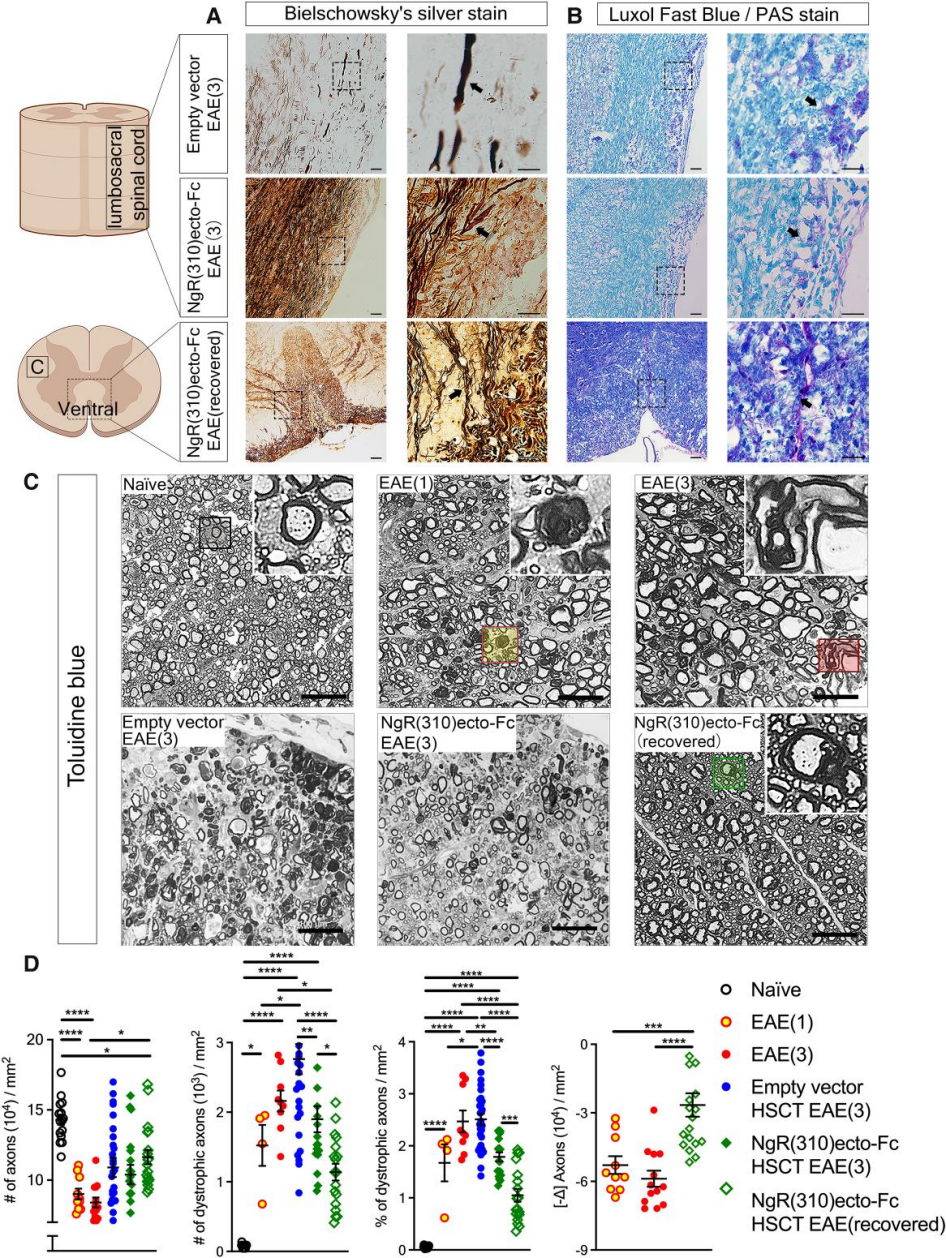
**Histological evidence of axonal degeneration and demyelination pathology in mice at the peak stage of EAE.** (**A**) Dystrophic and intact axons were revealed by Bielschowsky’s silver in white matter tracts of the spinal cord. Retraction bulbs and swollen and even transected axons (black arrows) can be identified at higher magnifications. Scale bar = 50 µm. (**B**) LFB-PAS staining demonstrated myelin debris engulfed by macrophages (black arrows) in white matter tracts from empty vector, NgR(310)ecto-Fc HSCT recipients at the peak of disease and clinically recovered mice. Scale bar = 50 µm. (**C**) Toluidine blue-stained semithin cross-sections from naïve, EAE clinical scores of 1 and 3, empty vector and NgR(310)ecto-Fc HSCT recipient animals at EAE clinical score 3 and clinically recovered animals. Insets showcase individual axons. (**D**) The number of total and dystrophic axons was quantified per mm^2^. The percentage of dystrophic axons and the delta change (−Δ) of axon dropouts were also calculated. *n* = 3 per group. One-way ANOVA with Tukey’s *post hoc* test. Data are represented as mean ± SEM. *P < 0.05, **P < 0.01, ***P < 0.001, ****P < 0.0001. Individual data points represent the analysis of axonal degeneration obtained from spinal cord white matter tracts (in toluidine blue-stained semithin cross sections) of experimental mice.

**Figure 7:**
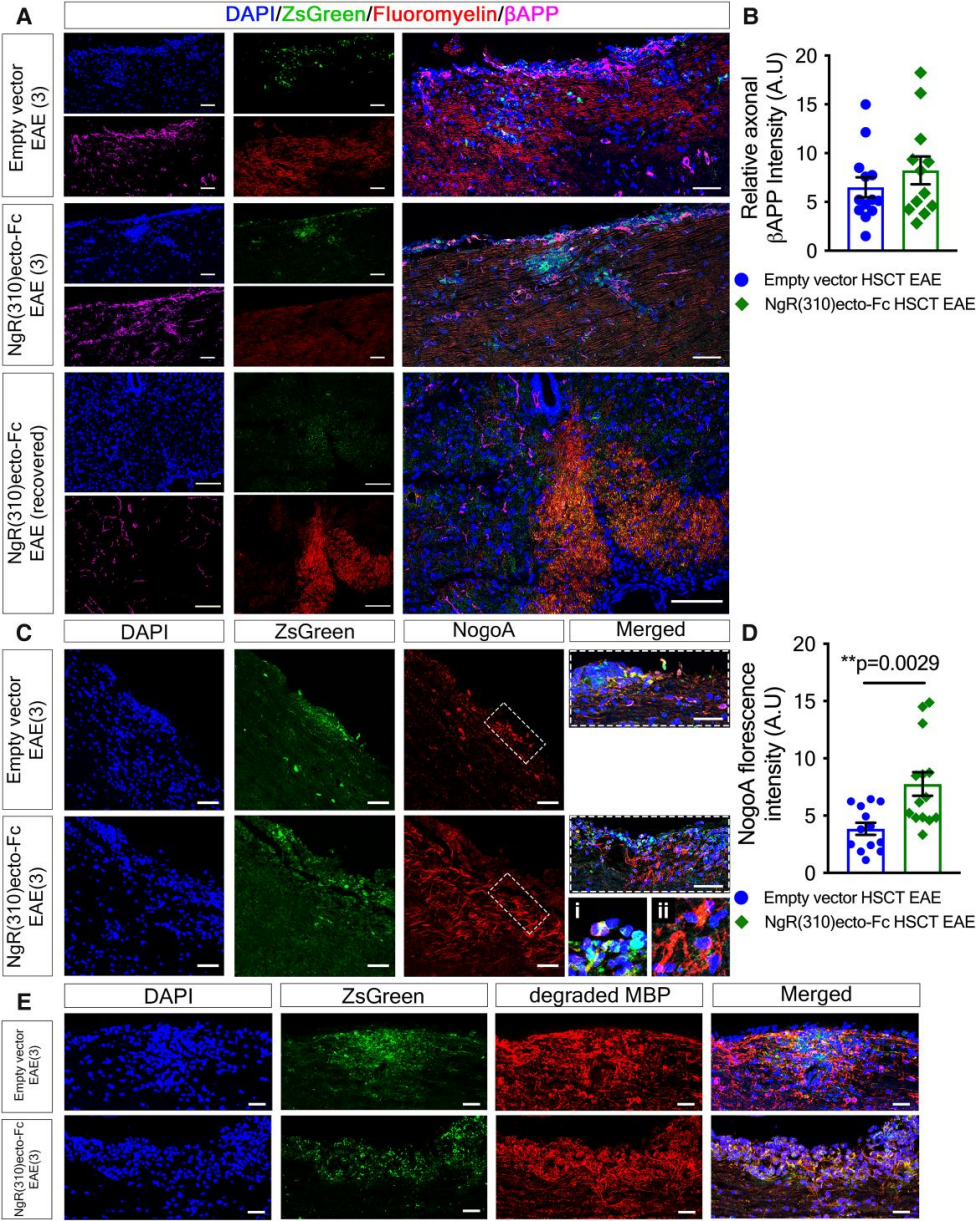
**Axonal damage, dystrophy (βAPP) and NogoA+ myelin debris engulfment exhibit variability within inflammatory lesions at the peak stage of the disease.** (**A**) Immunohistochemical analysis of ZsGreen (green), fluoromyelin (red) and βAPP (magenta) in the spinal cord of the empty vector, NgR(310)ecto-Fc HSCT and clinically recovered NgR(310)ecto-Fc HSCT mice. Scale bar = 100 μm. (**B**) The inflammatory lesion sites exhibited a high intensity but not significantly different levels of βAPP accumulation. *n* = 3 per group. Unpaired two-tailed *t*-test. Individual data points represent the analysis of fluorescence intensity obtained from inflammatory regions of spinal cord (longitudinal sections) of experimental mice. (**C**) Immunohistochemical analysis of ZsGreen (green) and NogoA (red) in the spinal cord of the empty vector, NgR(310)ecto-Fc HSCT and clinically recovered NgR(310)ecto-Fc HSCT mice. Scale bar = 100 μm. Hatched boxes depict close-up cellular populations within the lesion site. A representative image of (**C-i**) macrophages engulfing NogoA+ myelin debris and (**C-ii**) NogoA+ OPCs. (**D**) Increased fluorescent expression of NogoA in the ZsGreen+ inflammatory lesion areas in the NgR(310)ecto-Fc HSCT mice compared to the empty vector HSCT mice, suggesting the recruitment of oligodendroglial precursors (OPCs). *n* = 3 per group. Data are represented as mean ± SEM. Unpaired two-tailed *t*-test. **P < 0.01. Individual data points represent the analysis of fluorescence intensity obtained inflammatory regions of spinal cord (longitudinal sections) of experimental mice. (**E**) Immunofluorescence labelling of ZsGreen+ spinal cord infiltrates co-expressing degraded MBP (red), representing internalized myelin debris. Scale bar = 100 μm.

Both empty and NgR(310)ecto-Fc vector-transduced HSC recipient mice at the peak of EAE showed the classical pathological hallmarks of disease severity, including axonal degeneration and inflammatory demyelination (Fig. 6A and B). The numbers of dystrophic axons demonstrating morphological Wallerian degeneration were semi-quantitatively assessed on toluidine blue-stained semithin lumbar spinal cord cross-sections (Fig. 6C and D). Clinically recovered NgR(310)ecto-Fc HSCT recipients showed a 1.5-fold decrease in the number of dystrophic axons (Fig. 6D, the second graph) with increases in axonal numbers (Fig. 6D, the fourth graph) when compared to the peak of EAE, which are nearing the naïve state within the dorsal columns. Furthermore, the spinal cord of both transduced HSCs groups exhibited an accumulation of βAPP at the peak of EAE, indicating altered axonal transport^41,42^ associated with demyelination depicted by reduced fluoromyelin positivity (Fig. 7A and B). We identified increased NogoA expression within ZsGreen+ immune lineage cells (Fig. 7C and D), but importantly, in the NgR(310)ecto-Fc HSCT group at the peak of disease, indicating that NogoA, one of the integral MAIFs governing neurite outgrowth inhibition, was actively engulfed by ZsGreen+ cells in NgR(310)ecto-Fc-transduced groups (Fig. 7C-i). Furthermore, we also found elevated NogoA+ cells of an elongated morphology resembling pre-myelinating oligodendroglial precursor cells (OPCs) with branched processes (Fig. 7C-ii). These data confirmed that the demyelinated inflammatory lesions were consisted of immune-lineage cells which actively express NgR(310)ecto-Fc, implying the participation of cellular NogoA− NgR(310)ecto-Fc related therapeutic mechanism during EAE progression and corroborating the facilitated clearance of myelin debris illustrated in Figs. 3 and 4.

**Figure 8:**
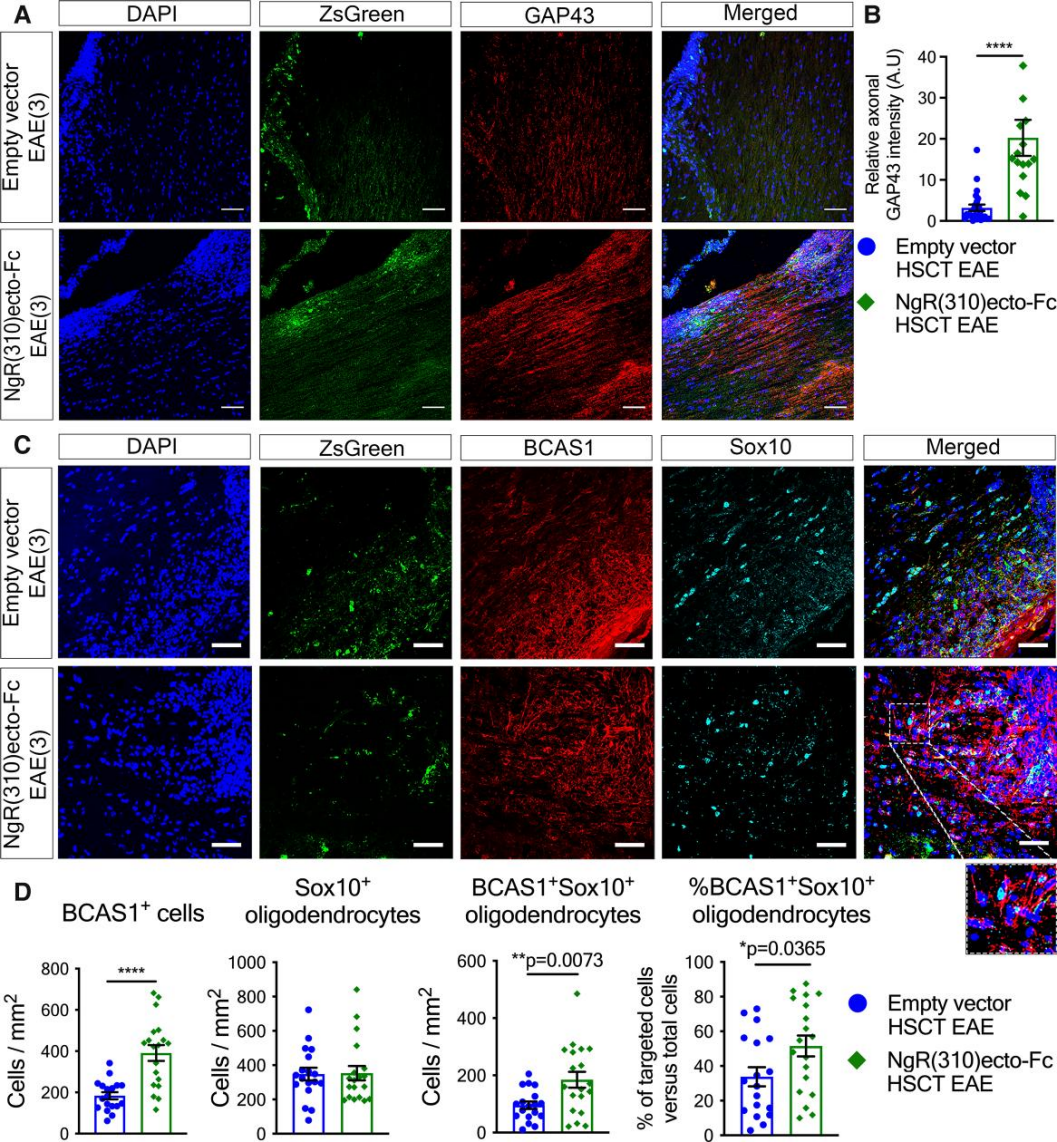
**Myelin structure is preserved along with an increased number of BCAS1+ remyelinating oligodendrocytes in NgR(310)ecto-Fc HSCT EAE mice.** (**A**) Immunohistochemical analysis of ZsGreen (green) and GAP43+ axonal area (red) within inflammatory lesion areas of spinal cord in the empty- and NgR(310)ecto-Fc HSCT animals at EAE clinical score of 3. Scale bar = 70 μm. (**B**) The relative increase in GAP43 intensity in the ZsGreen+ inflammatory lesions suggests enhanced axonal regeneration in the NgR(310)ecto-Fc HSCT-transduced animals. *n* = 3 per group. Data are represented as mean ± SEM. Mann–Whitney two-tailed *t*-test. ****P < 0.0001. Individual data points represent the analysis of fluorescence intensity obtained from inflammatory regions of spinal cord (longitudinal sections) of experimental mice. (**C**) Immunohistochemical analysis of ZsGreen (green), BCAS1 (red), and Sox10 (cyan) in the LSSC sections of the empty vector and NgR(310)ecto-Fc HSCT mice at EAE clinical score 3. Scale bar = 50 μm. (**D**) Quantification of BCAS1+ remyelinating and Sox10^+^ mature oligodendrocytes per inflammatory lesion area (mm^2^) in the empty vector- and NgR(310)ecto-Fc HSCT-transduced mice. The percentage of BCAS1+Sox10+ remyelinating oligodendrocytes was greater in NgR(310)ecto-Fc HSCT mice. *n* = 3 per group. Data are represented as mean ± SEM. Unpaired two-tailed *t*-test. *P < 0.05, **P < 0.01, ****P < 0.0001. Individual data points represent the analysis of cell numbers obtained inflammatory regions of spinal cord (longitudinal sections) of experimental mice.

Axonal NgR1 signalling is activated upon its high affinity binding with MAIFs expressed on myelin.^43-45^ In accordance with our hypothesis that the local production of NgR(310)ecto-Fc therapeutic protein could block NgR1-MAIF-dependent signalling, we showed that the HSC-transplanted recipient mice with NgR(310)ecto-Fc transduction, at the peak stage of EAE, exhibited potentiated growth-associated protein 43 (GAP43)^46^ expression levels at the peak stage of EAE, suggesting axon growth and plasticity or sprouting (Fig. 8A and B).

The observation of possible NogoA+ precursor cell mobilization and the enhanced axonal growth-related profile exhibited in our NgR(310)ecto-Fc vector-transduced HSC recipient group was further validated by measuring the number of bona-fide pre-myelinating OPCs. We explored the numbers of BCAS1+ and Sox10+ double-labelled OPCs which are commonly observed in chronic white matter lesions of multiple sclerosis patients.^47^ We observed 2-fold and 7-fold increases in NogoA and GAP43 expression, indicating recruitment of OPCs and axonal regeneration, respectively, as well as an increase in the number of BCAS1+ remyelinating oligodendrocytes which co-expressed Sox10 per inflammatory lesion area in NgR(310)ecto-Fc vector-transduced HSC recipient animals (Fig. 8C and D). Collectively, these data support the therapeutic property potential of the NgR(310)ecto-Fc protein as its delivery facilitates local axonal regeneration by antagonizing axonal NgR1-dependent signalling in dystrophic axons and subsequent recruitment of OPCs to lesion areas where the fusion protein was expressed, thereby potentially achieving repair.

### Remyelination of axons occurs in clinically recovered animals following transplantation with NgR(310)ecto-Fc vector-transduced HSCs

**Figure 9:**
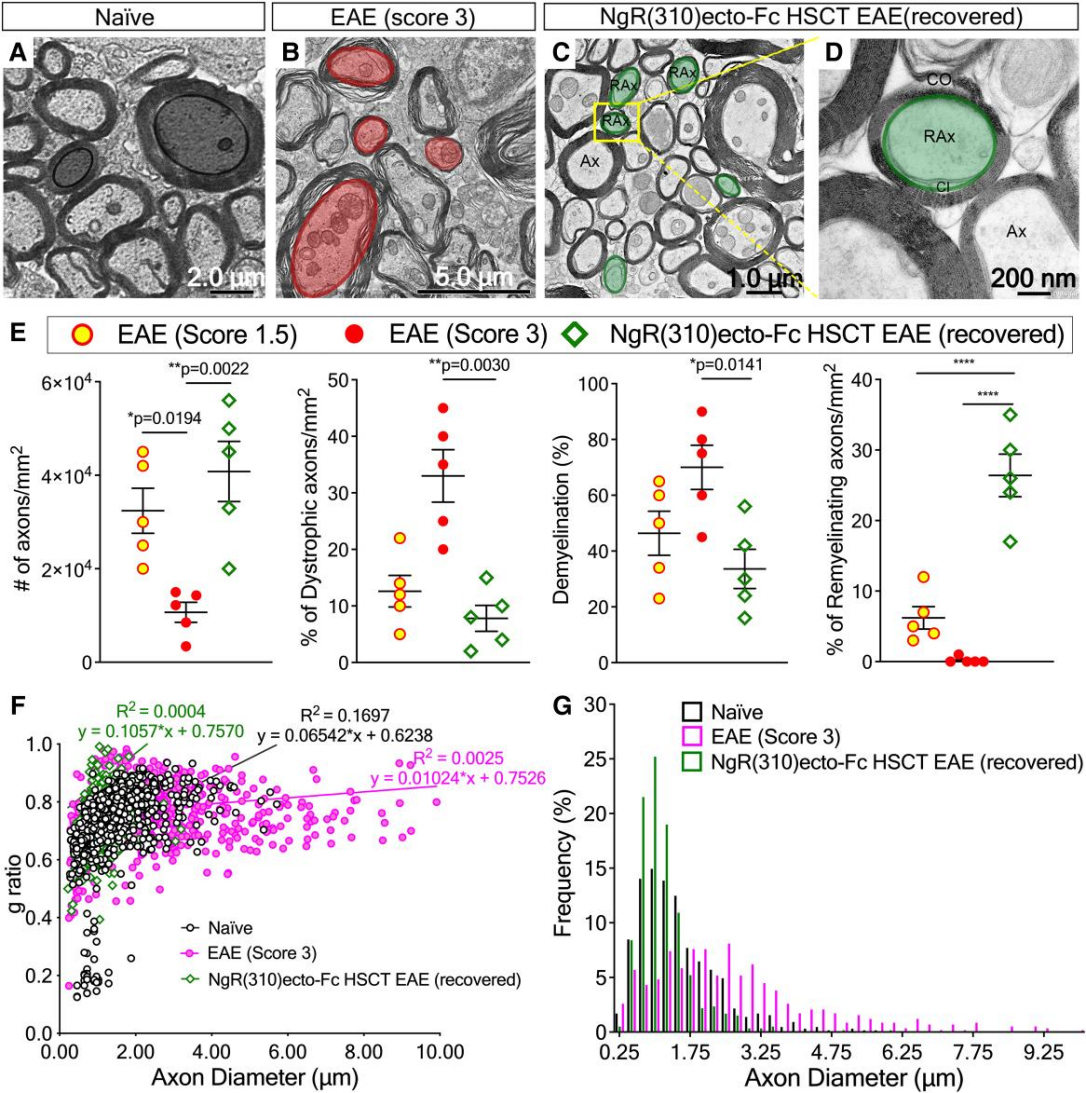
**Newly remyelinated axons are present in the LSSC white matter from clinically recovered NgR(310)ecto-Fc HSCT mice following the peak stage of disease.** (**A**–**D**) The dorsolateral white matter of lumbosacral spinal cord sections harvested from (**A**) naïve, (**B**) EAE clinical score of 3, and (**C**–**D**) the clinically recovered NgR(310)ecto-Fc HSCT recipients were assessed using electron microscopy. (**C**) Newly remyelinating (RAx) and intact axons with a compact myelin sheath (Ax) were observed. (**D**) Inner (CI) and outer spiral membranes (CO) of myelin ensheathing near axon where cytoplasm is later excluded following myelin compaction. (**E**) The number of total and dystrophic axons was quantified per mm^2^ in toluidine blue-stained semithin cross-sections from naïve, EAE clinical score of 1.5 and 3, and clinically recovered NgR(310)ecto-Fc HSCT mice. The percentage of demyelination and the number of newly remyelinated axons were also calculated. *n* = 3 per group. Data are represented as mean ± SEM. One-way ANOVA with Tukey’s *post hoc* test. *P < 0.05, **P < 0.01, ****P < 0.0001. Individual data points represent the analysis of myelinated axonal numbers obtained from toluidine blue-stained semithin cross sections of spinal cord white matter tracts of experimental mice. (**F**) A scatter plot displays *g* ratio of individual myelinated axons as a function of the respective axon calibre and the linear regression of the *g* ratio measurements for each group. (**G**) The percentile frequency of myelinated axons with respect to axon calibre at 0.25 μm intervals in lumbar white matter.

To assess whether increased OPC recruitment led to enhanced remyelination in the NgR(310)ecto-Fc-transduced HSCT group, we performed electron microscopic analysis. The characteristic ultrastructural features of increased myelinated axonal density, reduced dystrophy and newly remyelinating axons in which two-layered inner- and outer-spiral membranes were observed along with loose cytoplasm-filled membranes containing an uncompacted myelin sheath of limited lamellae (hypomyelinated), enclosing and surrounding many axons in the spinal cord dorsal columns of NgR(310)ecto-Fc HSCT recipient mice (Fig. 9A–E).

The majority of spinal cord axons were swollen, and their myelin sheaths were decompacted and degenerative in mice at an EAE clinical score of 3 (Fig. 9B). However, a 2-fold decrease in the percentage of demyelination were demonstrated in the clinically recovered animals compared to NgR(310)ecto-Fc vector-transduced HSC recipients at the peak stage of EAE (Fig. 9E, the third graph). Remyelinated fibres were observed in greater numbers among smaller calibre axons along with an increased *g* ratio (Fig. 9F). This enhanced remyelination process may be directly related to the increased population of BCAS1-labelled OPCs demonstrated in Fig. 8C and D. Indeed, the modulation of this cellular response could also be related to the endogenous microglial and astrocyte populations that were also potentiated in the spinal cords. In the NgR(310)ecto-Fc vector-transduced HSC recipient mice, we showed that there was a promotion of significant numbers of TREM2+ (Supplementary Fig. 6B and C), along with a reduced GFAP+C3+ ‘A1’ pro-inflammatory astrocytes present within inflammatory regions during the peak stage of EAE, whereas we observed an expanded GFAP+/S100A10+ ‘A2’ reactive astrocyte population^48,49^ present in the spinal cord peri-plaque white matter in the NgR(310)ecto-Fc vector-transduced HSC recipient mice that had recovered from the peak stage of EAE (Supplementary Fig. 7). These data correlated with reduced CSPG4+ immunolabelling at peak stage of disease in the NgR(310)ecto-Fc vector-transduced HSC recipient mice (Supplementary Fig. 7D). Collectively, these data strongly argue that targeted delivery of the NgR(310)ecto-Fc therapeutic protein to neuroinflammatory lesion sites following HSC transplantation with ensuing lineage differentiation can prevent ongoing neurological decline and may enhance neurorepair through axon regrowth and remyelination.

## Discussion

In the present study, we developed a superior and promising therapeutic strategy utilizing migratory immune cells to deliver NgR(310)ecto-myc-Fc fusion protein directly to disseminated inflammatory demyelinating lesion sites by transplanting NgR(310)ecto-Fc vector-transduced HSCs into the EAE animal model of multiple sclerosis. In this proof-of-principle study, we found that there were no alterations in the functional capacity of transduced HSCs in the recipient mice, as demonstrated by successful cell engraftment and chimerism in the periphery. Upon EAE induction following immunization with MOG_35–55_, HSCs transduced with the NgR(310)ecto-Fc vector and transplanted into recipient female mice exhibited reduced clinicopathological characteristics associated with axonal pathology. Importantly, mice from the NgR(310)ecto-Fc vector-transduced HSCT group displayed complete recovery of the clinical symptoms of EAE.

Taking advantage of activated immune cells that migrate into the CNS during inflammatory demyelinating disease progression, we observed no overt modulation in the immunopathogenesis of EAE following the transplantation of either empty vector- or NgR(310)ecto-Fc vector-transduced HSCs in wildtype recipient mice. However, we identified inflammatory infiltrates expressing and secreting NgR(310)ecto-Fc protein, which were predominantly derived from CD11b+ macrophages within the spinal cord lesions at the peak stage of EAE clinical score 3. In fact, it was shown that lentivirally modified HSCs for gene therapy can be utilized in myeloid lineage cells and enter the CNS to replace tissue-resident macrophages and microglial-like cells, demonstrating therapeutic efficacy as an alternative to enzyme replacement therapy for inherited neurometabolic diseases in human trials.^50-54^ Collectively, our paradigm-shifting data suggest that our lentivirus-based NgR(310)ecto-Fc-transduced HSC transplantation is clinically translatable.

Furthermore, we observed the presence of ZsGreen lineage CD11b+ myc+ macrophages, which may phagocytose and actively remove NgR(310)ecto-Fc-bound myelin debris. Enhanced clearance of myelin debris is now becoming recognized as a prominent biological mechanism for promoting axonal preservation, regeneration, and remyelination.^55^ The role of myelin-clearing phagocytic macrophages or CNS-resident microglia in local immunosuppression has also been implicated in recent investigations.^56-58^ Studies have revealed the presence of myelin-laden macrophages/microglia releasing anti-inflammatory molecules in demyelinating multiple sclerosis lesions and the suppression or switching of detrimental phenotype of classically activated macrophages/microglia to anti-inflammatory cells followed by myelin debris clearance.^59,60^ Immunogold-labelled electron microscopic analyses revealed an enhanced uptake of the myc+ protein-laden macrophages within lesions of the NgR(310)ecto-Fc-transduced HSC-transplanted mice with NogoA+ and degraded MBP+ protein expression present within these cells. These data support the notion that the local lesioned production of NgR(310)ecto-Fc is expediting the engulfment of myelin proteins, probably from debris.

Our results suggest that NgR(310)ecto-Fc proteins did not affect inflammatory cell infiltrates and CNS pathology in EAE since there were no significant differences in the levels of inflammatory demyelination and axonal degeneration detected by immune cell infiltration throughout and βAPP accumulation, respectively, in the NgR(310)ecto-Fc-transduced mice when compared to the empty vector control mice. Furthermore, the number of dystrophic axons undergoing Wallerian degeneration was significantly reduced in the clinically recovered mice with vastly elevated small calibre fibres present, suggesting the recovery of axons after profound neuroinflammation. Enhanced axonal regeneration in NgR(310)ecto-Fc-transduced HSCT recipients was implicated by an increased intensity of GAP43 in the axons, which is one of the common histological characteristics indicating regenerative axons in the CNS since GAP43 is associated with neurite regrowth and plasticity.^46^ These results suggest that secreted NgR(310)ecto-Fc proteins contributed to axonal regeneration by limiting degenerative axonal NgR1-dependent signalling and promoted the phagocytic activity of macrophages by removal of NgR(310)ecto-Fc-bound myelin debris.

Of great significance for this reparative paradigm, our electron microscopy analysis showed significantly increased *g* ratios in the clinically recovered NgR(310)ecto-Fc-transduced HSC recipients. This outcome in the recovered mice was further substantiated in the diseased spinal cords of NgR(310)ecto-Fc-transduced HSC recipient mice that showed greater numbers of BCAS1+ exhibited mobilization of remyelinating oligodendrocytes. These observations support the therapeutic roles of NgR(310)ecto-Fc protein to repair denuded axons by enhancing myelin restoration and support the recent findings of the NgR(310)ecto-Fc therapeutic fusion protein in a stroke model.^61^ Furthermore, the cluster of NogoA+ OPCs localized to demyelinated regions, enriched with ZsGreen+ and myc+ macrophages within the NgR(310)ecto-Fc vector-transduced HSC-transplanted mice, may further support an attractive/mobilization role for NogoA+ OPCs when exposed to local NgR(310)ecto-Fc at lesion sites.

It has been suggested that CNS myelin debris may impede or are potent endogenous inhibitory cues for OPC recruitment and maturation.^62^ Importantly, NogoA expression is not the only myelin-bound inhibitory cue involved in the development of myelin structure (i.e. internodes) formed by oligodendrocytes since blocking NogoA showed no changes in internodal properties.^63^ This may provide an explanation for unsuccessful anti-NogoA therapy that resulted in low therapeutic efficacy. Despite concerns pertaining to the stability and safety of lentiviral vectors, studies using lentivirus-dependent gene therapy have previously shown insignificant immune responses against lentiviral vectors in animal models of neurological disorders.^64^ In addition, clinical applications of lentivirus-transduced HSCs showed an acceptable safety profile. Follow-up studies for treating inborn errors of metabolic diseases that affect the CNS, such as X-linked adrenoleukodystrophy, metachromatic leukodystrophy and Wiskott–Aldrich syndrome, have all reported excellent outcomes.^65-71^ Specific concerns related to random integration of lentiviral vectors have been reported, with unexpected serious adverse events of acute myeloid leukaemia during the LentiGlobin (Bluebird Bio) trial for sickle cell disease.^72^ However, this was later reported to be unrelated to the therapeutic lentiviral vector.^72^ Nevertheless, in a future study, one can devise a gene editing-based integration of NgR(310)ecto-Fc into a safe harbour locus for safer gene transfer.^73,74^

## Conclusion

In summary, the current study suggests a potential therapeutic approach for progressive multiple sclerosis by targeting axonal NgR1-dependent signalling, which highlights its inhibitory role in neurodegeneration. This outcome has profound implications for the development of novel therapeutic approaches for progressive multiple sclerosis. Since many biological therapeutic candidates in the drug development pipeline have failed to show high therapeutic efficacy, as these agents cannot cross the blood–brain barrier or directly target disseminated multiple sclerosis lesions within the CNS,^75^ such a novel approach may provide a potential solution for neuroprotection and repair paradigms for ∼2.8 million individuals worldwide living with the disease.^76^ Here, we report the therapeutic potential of transplanted HSCs transduced with NgR(310)ecto-Fc, reporting its capacity to drive axonal regeneration and, most importantly, remyelination in a model of multiple sclerosis-like disease, with complete recovery from neurological decline. Our data also suggest that NgR(310)ecto-Fc-transduced HSC-derived phagocytic macrophages play an important part in the delivery of the fusion peptide and may exert an alternatively activated phenotype that is anti-inflammatory and capable of switching its polarization to eliminate NgR(310)ecto-Fc-bound myelin debris. This expedited clearance of myelin debris may indeed be modifying the extracellular milieu to enhance axonal preservation, regeneration and remyelination. This study has provided proof-of-principle data for future development in preclinical models and the possibility for future clinical trials.

## Supplementary material

Supplementary material is available at *Brain Communications* online.

## Supplementary Material

fcad108_Supplementary_Data

## Abbreviations

AD =: axial diffusivity
ADC =: apparent diffusion coefficient
ALS =: amyotrophic lateral sclerosis
Arg1 =: arginase 1
BBB =: blood–brain barrier
BCA =: bicinchoninic acid protein
BCAS1 =: brain enriched myelin associated protein 1
BSA =: bovine serum albumin
CFA =: complete Freund’s adjuvant
DMEM =: Dulbecco's Modified Eagle Medium
DTI =: diffusion tensor imaging
EAE =: experimental autoimmune encephalomyelitis
EBSS =: Earle’s balanced salt solution
ECL =: enhanced chemiluminescence
empty vector HSCT EAE =: recipient female mice transplanted with haematopoietic stem cells transduced by lentiviral vectors only carrying ZsGreen reporter induced with experimental autoimmune encephalomyelitis
FA =: fractional anisotropy
FBS =: foetal bovine serum
FISH =: fluorescent *in situ* hybridization
Flt3 =: ligand FMS-like tyrosine kinase 3 ligand
GAP43 =: growth-associated protein 43
GFAP =: glial fibrillary acidic protein
GM =: grey matter
HEK293T =: human embryonic kidney 293T cell
HRP =: horseradish peroxidase
HSCT =: haematopoietic stem cell transplantation
IgG =: immunoglobulin G
IL-3 =: interleukin 3
IL-6 =: interleukin 6
iNOS =: inducible nitric oxide synthase
IP =: immunoprecipitation
IR =: inflammatory region
LFB-PAS =: luxol fast blue and periodic acid–Schiff staining
LRR =: leucine rich repeat
LSSC =: lumbosacral spinal cord
MAG =: myelin-associated glycoprotein
MAIF =: myelin-associated inhibitory factor
MBP =: myelin basic protein
mf-VEP =: multifocal field visual evoked potential measures
mock HSCT EAE =: recipient female mice transplanted with non-transduced haematopoietic stem cells induced with experimental autoimmune encephalomyelitis
MOG =: myelin oligodendrocyte glycoprotein
MOI =: multiplicity of infection
MRP14 =: myeloid-related protein 14
NfL =: neurofilament light chain
NgR =: Nogo receptor
NgR(310)ecto-Fc =: Nogo receptor(1-310) ectodomain-myc-Fc chimeric protein
NgR(310)ecto-Fc HSCT EAE =: recipient female mice transplanted with haematopoietic stem cells transduced by lentiviral vectors carrying NgR(310)ecto-Fc and ZsGreen reporter induced with experimental autoimmune encephalomyelitis
OMgp =: oligodendrocyte myelin glycoprotein
OPC =: oligodendroglial precursor cell
PBS =: phosphate buffered saline
PFA =: paraformaldehyde
PPWM =: peri-plaque white matter
PVDF =: polyvinylidene fluoride
RD =: radial diffusivity
ROI =: region of interest
SCF =: stem cell factor
SCoRe =: spectral confocal reflection microscopy
SEM =: standard error of the mean
SIMOA =: single molecule array
TBST =: Tris-based saline with 0.1% v/v Tween-20
TEM =: transmission electron microscopy
TREM2 =: triggering receptor expressed on myeloid cells 2
WM =: white matter
βAPP =: beta-amyloid precursor protein
