# Supplementary material for: Nogo receptor-Fc delivered by haematopoietic cells enhances neurorepair in a multiple sclerosis model
Source: Brain Commun. 2023 Apr 4;5(2):fcad108. doi: 10.1093/braincomms/fcad108 (PMC10116608; doi:10.1093/braincomms/fcad108)
Supplement: fcad108_Supplementary_Data [file fcad108_supplementary_data.pdf]

Supplementary Methodology

Lentivirus generation via HEK 293T cell transfection

Human embryonic kidney (HEK) 293T cells were used to produce functional lentiviral vectors from packaging plasmids and either pLVX-EF1α-IRES-ZsGreen1 (from here referred to as empty vector) or pLVX-EF1α-IRES-ZsGreen1-NgR(310)ecto-myc-Fc (from here referred to as NgR(310)ecto-myc-Fc/ NgR(310)ecto-Fc) plasmids. A day prior to transfection, HEK 293T cells were seeded into 10 cm tissue culture dishes with 5×10^6 cells per dish in 10 mL antibiotic-free HEK 293T complete medium (10% tetracycline-free FBS, 1% L-glutamine in DMEM). Two hours prior to transfection, the medium was aspirated and replaced with 6 mL fresh antibiotic-free HEK 293T complete medium. The transfection reaction for each dish was prepared with 3.2 µg psPAX2 (Addgene #12260), 0.4 µg pMD2.G (Addgene #12259), 3.2 µg empty plasmid or NgR(310)ecto-myc-Fc plasmid in 40 µL dH_2O with 160 µL DMEM (Life technologies) and 24 µL FuGene-HD transfection reagent (Promega), gently mixed and incubated for 30 minutes at room temperature. The transfection reaction was then added dropwise to evenly cover the HEK 293T cells which were subsequently incubated at 37 °C for 72 hours to allow for empty and NgR(310)ecto-Fc vector production. The empty or NgR(310)ecto-Fc vector-containing supernatant was then collected and filtered through a 0.45 µm filter prior to concentration. Lentiviral vector containing transfection supernatant was concentrated by ultracentrifugation. The supernatant was added to V-bottom canonical tubes (Beckman Coulter) and centrifuged in a Beckman Coulter Optima L-80 floor centrifuge with SW-28 rotor at 20,000 rpm for 140 minutes at 16 °C. The supernatant was then discarded, and the lentiviral vector pellet was resuspended in 50 µL DPBS (Life technologies) with 0.5% w/v bovine serum albumin (BSA; Sigma-Aldrich) and allowed to incubate for 30 minutes on ice. The samples were then resuspended again and pooled for storage at -80 °C.

Quantification of vectors via HeLa transduction

To ascertain the quantity of functional lentiviral vectors produced, a virus titration was performed in Henrietta Lacks (HeLa) cells. A day prior to transduction, HeLa cells were seeded at 1×10^5 cells per well, in 6 well plates with DMEM complete medium (10% FBS, 1% Pen/Strep, 1% L-glutamine, Life technologies). Spin infection (90 minutes, 32 °C, 1200 xg) was performed in a 2 mL per well volume with serial lentiviral vector dilutions, where 5.0×10^{-3}, 5.0×10^{-4}, 5.0×10^{-5}, 5.0×10^{-6}, and 5.0×10^{-7} mL of empty vector or, of NgR(310)ecto-Fc vector was added, along with 8 µg/mL polybrene (Sigma-Aldrich). Post-spin infection, cells were incubated for 24 hours before replacement of medium and further incubation. After 72 hours post transduction, cells were analyzed for construct expression by flow cytometry.

Virus titer was calculated as

$$\frac{\text{Number of cells at seeding} \times \text{\% of Zsgreen+ cells}}{\text{volume of virus } (\mu\text{L})} = \text{Virus titer}/\mu\text{L}.$$

Flow cytometry

The animals that reached clinical score 3 were euthanized by CO_2 inhalation and perfused with 1x MT-PBS, then peripheral blood, inguinal lymph nodes, and spleen were collected and prepared as follows for flow cytometric analysis. Briefly, red blood cell (RBC) lysis was performed by addition of 1 mL pre-warmed RBC lysis buffer and incubating for 2 minutes at room temperature. The lysis was then quenched with 5 mL fluorescence activated cell sorting (FACS) buffer and centrifuged for 5 minutes at 1500 rpm, then resuspended in FACS buffer. The cell suspensions were centrifuged and resuspended in cold wash buffer before being aliquoted into 5 mL flow cytometry tubes. Cells were incubated with primary antibodies (Supplementary Table 2) for 30 minutes on ice, then washed with 1 mL of wash buffer and centrifuging for 5 minutes. These samples were analyzed by flow cytometry to determine

chimerism by passage through a LSR II (BD Biosciences), using FACS Diva Software (BD Biosciences) for acquisition and FlowLogic version 700.1A. for post-analysis.

Sandwich ELISA

In order to detect the secreted NgR(310)ecto-Fc fusion protein in vivo post-transplantation, the anti-myc antibody (Cell signaling technologies) was used as a capture antibody and anti-NgR antibody (R&D systems) served as the detection antibody. A 96-well plate was coated with an anti-myc antibody at a concentration of 1 μg/mL in carbonate/bicarbonate buffer (pH 9.6) and incubated overnight at 4 °C. The plate was washed twice with PBS, followed by blocking the remaining protein-binding sites in the coated wells using blocking buffer (5% w/v BSA in PBS) overnight at 4 °C. Each sample was run in triplicates to ensure accuracy. 1 μg of full-length NgR-Fc peptide served as a positive control. The samples were diluted in the final volume of 100 μL and then incubated for 90 minutes at 37 °C. Subsequently, the plate was incubated with detection antibody for 2 hours at room temperature. The plate was washed four times with PBS. The secondary antibody (anti-goat HRP-conjugated, Supplementary Table 2) was diluted in blocking buffer and incubated for 2 hours, at room temperature, followed by four washes with PBS. The substrate solution, ABTS, was added to each well and the plate was read at 416 nm on the Thermo Multiskan microplate reader.

Fluorescence in situ hybridization (FISH)

OCT-embedded tissue sections were prepared the same as immunohistochemistry. The slides were pre-treated with 2.5% pepsin solution (80 mg/ mL) in 0.01N HCL for 30 minutes, then rinsed in 70% ethanol for 30 seconds and air-dried to achieve digestion. Subsequently, slides were dehydrated through an ethanol gradient (70%, 85%, and 100% for 2 minutes each). The slides were immersed in a denaturation buffer to separate DNA strands and incubated at 73 °C

for 5 minutes. The slides were washed with ethanol gradient (70%, 85%, and 100% for 2 minutes each) and placed on a slide warmer at 45 °C until dry. 10 μL of DNA probe mixture (2 μL DNA probe + 8 μL hybridization buffer; Empire Genomics) was prepared per slide (and pre-warmed at 73 °C for 5 minutes. The probe mixture was placed on ice for 2 minutes then incubated at 37 °C for 15 minutes). The slides were incubated overnight with DNA probe mixture in a humidified chamber with humidity control (50% formamide, 2 x SSC) at 37 °C. The slides were washed with pre-warmed wash solution 1 (0.3% v/v igepal, 0.4 x SSC in deionized water) at room temperature for 2 minutes, followed by rinsing with wash solution twice (0.1% v/v igepal, 2 x SSC in deionized water) at room temperature for 1 minute. The samples were stained with DAPI and mounted with anti-fade mounting medium (Life technologies).

Spectral confocal microscopy of reflectance imaging (SCoRe)

The visualisation of reflectance of myelinated axons and myelin debris can be acquired by Nikon A1R confocal microscopy, revealing the multipronged information of myelin structure and compaction. 40 μm thick slices of longitudinal spinal cord section directly mounted by fluorescent mounting medium were scanned using spectral confocal microscopy of reflectance (ScoRe) imaging. Only one laser beam was allowed to provide reflectance illumination of myelin. The first dichroic mirror was altered to the transmitted reflectance mirror BS20/80. All light path detectors were set up to “through”.

Supplementary Tables

Supplementary Table 1. Incidence table of survival in MOG_{35-55} induced EAE disease in animals

	EAE control	Mock HSCT EAE control	Empty vector HSCT EAE	NgR(310)ecto-Fc HSCT EAE
**Survival post-transplantation**	26/26 (100%)	13/17 (76.5%)	18/26 (61.54%)	21/27(77.78%)
**Mortality post-transplantation**	0	4	7	6
**Sudden death post-transplantation**	0	0	0	0
**EAE incidence**	25/26	9/13	10/18	13/21
**Mean day of onset of diseased mice only**	14.60 ± 0.42	12.78 ± 0.36	16.10 ± 0.53	15.69 ± 0.61
**Maximum score**	3	3	3	3
**Median score**	1.74	2.75	1.83	0.7

Supplementary Table 2. Key reagents and resources

Immunohistochemistry							
**Primary antibody**	**Vendor**	**Catalogue #**	**Dilution factor**	**Secondary antibody**	**Vendor**	**Catalogue #**	**Dilution factor**
Rat anti-CD11b	BD biosciences	553308	1:200	Goat anti-rat Alexa Fluor 647	Invitrogen	A-21247	1:500
Rabbit anti-myc	CST	2278	1:200	Goat anti-rabbit Alexa Fluor 555	Invitrogen	A-21428	1:500
Mouse anti-myc (9B11)	Cell signalling	2276S	1:100	Goat anti-mouse Alexa Fluor 647	Invitrogen	A-21236	1:200
Rabbit anti-NogoA	Millipore	AB5888	1:200	Goat anti-rabbit Alexa Fluor 555	Invitrogen	A-21428	1:200
Rabbit anti-degraded MBP	Millipore	AB5864	1:2000	Goat anti-rabbit Alexa Fluor 555	Invitrogen	A-21428	1:2000
Mouse anti-MRP14	Sigma Aldrich	MABF276	1:200	Goat anti-mouse Alexa Fluor 647	Invitrogen	A-21236	1:200
Rat anti-CD206	Biolegend	141701	1:200	Goat anti-rat Alexa Fluor 555	Invitrogen	A-21434	1:200
Alexa Fluor 555-conjugated Fluromyelin	Life technologies	F34652	1:100	N/A			
Mouse anti-βAPP	Thermo fisher	MA125489	1:200	Goat anti-mouse Alexa Fluor 647	Invitrogen	A-21236	1:500

Rabbit anti-GAP43	Abcam	ab75810	1:1000	Goat anti-rabbit Alexa Fluor 647	Invitrogen	A-32733	1:500
Mouse anti-BCAS1	Santa Cruz	sc-393808	1:200	Goat anti-mouse Alexa Fluor 555	Invitrogen	A-21236	1:500
Goat anti-Sox10	R&D systems	AF2864	1:200	Donkey anti-goat Alexa Fluor 647	Invitrogen	A-32849	1:500
Rat PE-conjugated anti-cKit	eBioscience	12117182	1:200		N/A		
Rat anti-CD19	Biolegend	115552	1:200	Goat anti-rat Alexa Fluor 555	Invitrogen	A-21434	1:200
Hamster PerCP-Cy5.5-conjugated anti-CD3e	BD bioscience	551163	1:200		N/A		
Rabbit anti-Iba1	Novus Biologicals	NBP2-16908	1:200	Goat anti-rabbit Alexa Fluor 555	Invitrogen	A-21428	1:500
Rabbit anti-TREM2	Thermo fisher	PA5-87933	1:100	Goat anti-rabbit Alexa Fluor 555	Invitrogen	A-21428	1:200
Alexa Fluor 647-conjugated anti-GFAP	BD bioscience	560298	1:50		N/A		
Rabbit anti-C3	Biorbyt	Orb453141	1:200	Goat anti-rabbit Alexa Fluor 555	Invitrogen	A-21428	1:200
Rabbit anti-S100A10	Thermo fisher	PA5-95505	1:500	Goat anti-rabbit Alexa Fluor 555	Invitrogen	A-21428	1:500

				DAPI	Thermo fisher	62248	1:1000

Immunopanning / Immunoprecipitation / Western blot

Primary antibody	Vendor	Catalogue #	Dilution/ Concentration	Secondary antibody	Vendor	Catalogue #	Dilution factor
Goat anti-rabbit IgG (H+L)	Life technologies	81-1620	10mg/ml	Rabbit anti-mouse CD11b	Sapphire Bioscience	NB110-89474	1:10
Mouse anti-myc	Millipore	05724	1:100		N/A		
Rabbit anti-Nogo66	Millipore	AB5888	1:1000	HRP-conjugated Goat anti-Rabbit	Sigma Aldrich	401353	1:2000
Rabbit anti-MAG	Cell signalling	12275	1:1000	HRP-conjugated Goat anti-Rabbit	Sigma Aldrich	401353	1:2000
Rat anti-mNgR1	R&D systems	MAB1659	1:1000	HRP-conjugated Goat anti-Rat	Invitrogen	31470	1:2000
Goat HRP-conjugated anti-mouse IgG-Fc	Abcam	Ab97265	1:1000		N/A		
Mouse anti- αtubulin	Millipore	MAB1637	1:1000	HRP-conjugated Goat anti-Mouse	Invitrogen	31430	1:2000
Mouse anti-βactin	Thermo fisher	26634	1:1000	HRP-conjugated Goat anti-Mouse	Invitrogen	31430	1:2000

Flow cytometry

Primary antibody	Vendor	Catalogue #	Dilution factor
Rat PE-conjugated anti-B220	BD biosciences	553090	1:200
Hamster APC-conjugated anti-CD11c	BD biosciences	550261	1:50
Hamster PerCP-Cy5.5-conjuated-anti-CD3e	BD biosciences	551163	1:50
Rat biotinylated-anti-CD11b	BD biosciences	557395	1:50
Rat PE-Cy7-conjugated anti-Sca-1	eBioscience	25598181	1:400
Streptavidin-APC	eBioscience	17431782	1:300

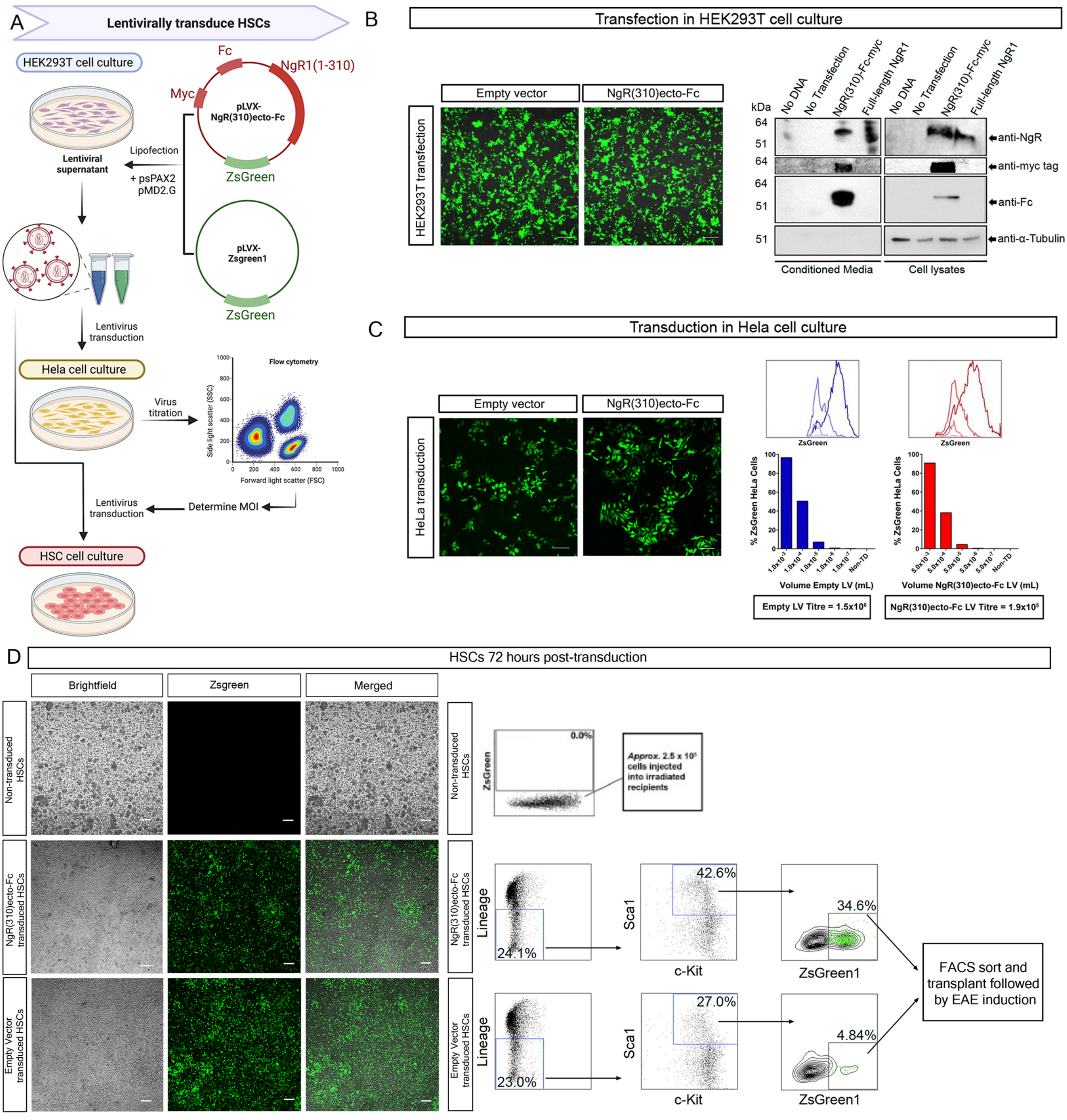
A
Lentivirally transduce HSCs
HEK293T cell culture
Lentiviral supernatant
Lipofection
+ psPAX2
pMD2.G
Fc
NgR1(1-310)
Myc
pLVX-NgR(310)ecto-Fc
ZsGreen
pLVX-Zsgreen1
ZsGreen
Lentivirus transduction
Hela cell culture
Virus titration
Flow cytometry
Side light scatter (SSC)
Forward light scatter (FSC)
Determine MOI
Lentivirus transduction
HSC cell culture
B
Transfection in HEK293T cell culture
Empty vector
NgR(310)ecto-Fc
HEK293T transfection
kDa
No DNA
No Transfection
NgR(310)-Fc-myc
Full-length NgR1
64
51
anti-NgR
anti-myc tag
anti-Fc
anti-α-Tubulin
Conditioned Media
Cell lysates
C
Transduction in Hela cell culture
Empty vector
NgR(310)ecto-Fc
HeLa transduction
ZsGreen
% ZsGreen HeLa Cells
Non-TD
Volume Empty LV (mL)
Volume NgR(310)ecto-Fc LV (mL)
Empty LV Titre = 1.5x10⁶
NgR(310)ecto-Fc LV Titre = 1.9x10⁵
D
HSCs 72 hours post-transduction
Brightfield
Zsgreen
Merged
Non-transduced HSCs
NgR(310)ecto-Fc transduced HSCs
Empty Vector transduced HSCs
ZsGreen
0.0%
Approx. 2.5 x 10⁵ cells injected into irradiated recipients
Lineage
24.1%
Sca1
c-Kit
42.6%
34.6%
ZsGreen1
23.0%
27.0%
4.84%
FACS sort and transplant followed by EAE induction

Supplementary Figure 1

Successful transfection and transduction of lentivirus carrying NgR(310)ecto-Fc fusion protein.

(**A**) Schematic workflow diagram performed to achieve lentiviral transduction in HSCs. (**B**) Lentiviral envelope and packaging plasmids in conjunction with transfer plasmids carrying either ZsGreen alone or NgR(310)ecto-Fc fusion protein and ZsGreen was used to generate lentivirus in HEK 293T cells following lipofection. Scale bar = 100 μm. Pre-immunoprecipitation data with 5% input of protein from in vitro conditioned media and cell lysates from the transfected 293T HEK cells, followed by immunoprobing all western blot transfer membranes with anti-NgR, anti-myc tag, anti-Fc and anti- α-tubulin antibodies. (**C**) Both packaging lentivirus carrying ZsGreen alone (empty vector) and NgR(310)ecto-Fc fusion protein was titrated through the transduction of HeLa cells. Transduction was conducted following a serial dilution range of different viral titres**.** Scale bar = 100 μm. 72 h post-transduction, the percentage of HeLa cells as ZsGreen-positive were determined by flow cytometry. (**D**) 72h post-transduction, empty vector and lentivirus carrying NgR(310)ecto-Fc was successfully transduced into lineage-depleted HSCs isolated from male C57Bl6 donor mice at multiplicity of infection of 50. Scale bar = 100 μm. The expression of c-Kit, Sca1 and ZsGreen of lineage-negative enriched cells was determined as the percentage of these markers from the total cells identified through flow cytometry.

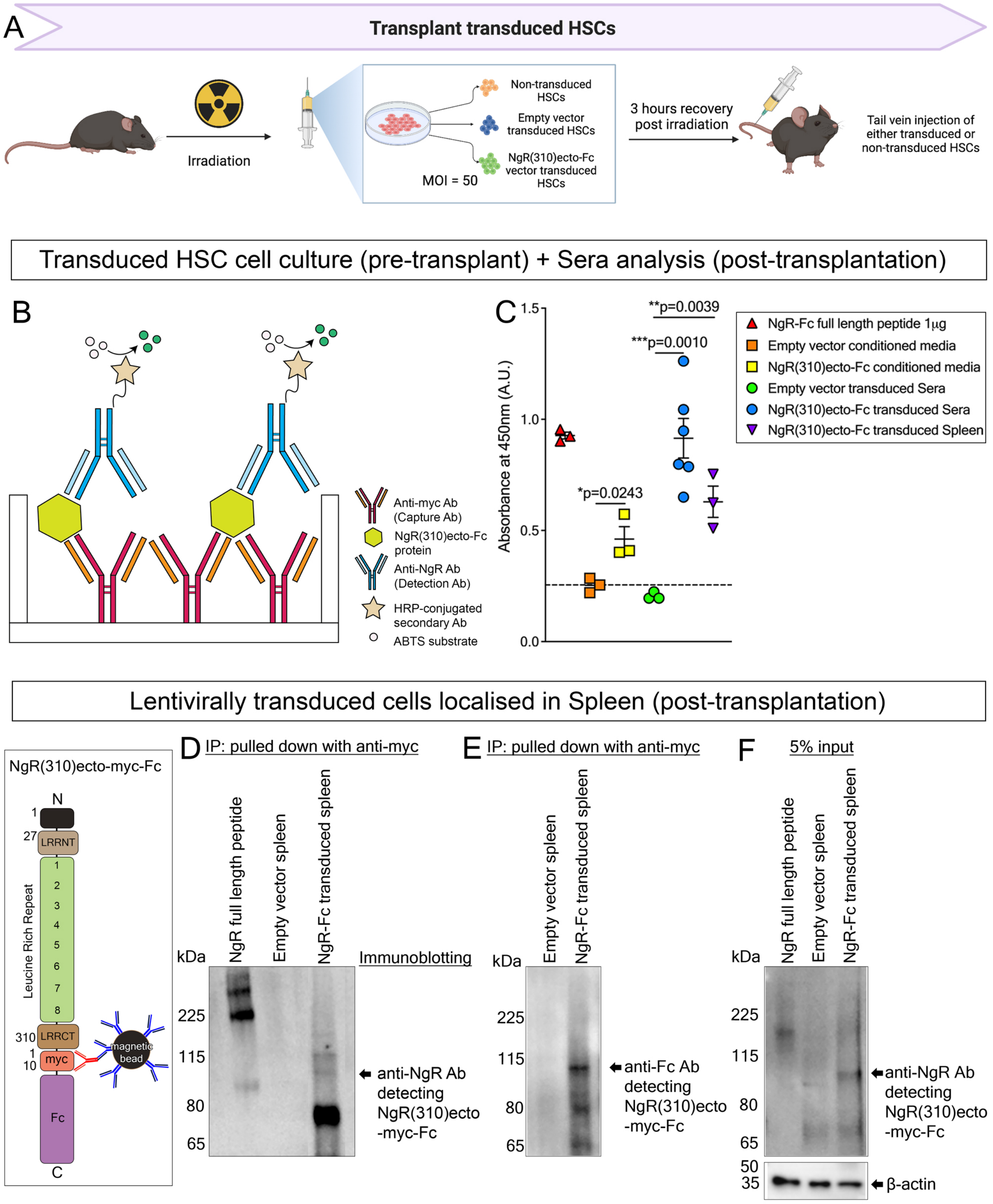

A
Transplant transduced HSCs
Irradiation
Non-transduced HSCs
Empty vector transduced HSCs
NgR(310)ecto-Fc vector transduced HSCs
MOI = 50
3 hours recovery post irradiation
Tail vein injection of either transduced or non-transduced HSCs
Transduced HSC cell culture (pre-transplant) + Sera analysis (post-transplantation)
B
Anti-myc Ab (Capture Ab)
NgR(310)ecto-Fc protein
Anti-NgR Ab (Detection Ab)
HRP-conjugated secondary Ab
ABTS substrate
C
Absorbance at 450nm (A.U.)
1.5
1.0
0.5
0.0
**p=0.0039
***p=0.0010
*p=0.0243
NgR-Fc full length peptide 1μg
Empty vector conditioned media
NgR(310)ecto-Fc conditioned media
Empty vector transduced Sera
NgR(310)ecto-Fc transduced Sera
NgR(310)ecto-Fc transduced Spleen
Lentivirally transduced cells localised in Spleen (post-transplantation)
NgR(310)ecto-myc-Fc
N
1
27
LRRNT
Leucine Rich Repeat
1
2
3
4
5
6
7
8
310
LRRCT
1
10
myc
magnetic bead
Fc
C
D
IP: pulled down with anti-myc
kDa
NgR full length peptide
Empty vector spleen
NgR-Fc transduced spleen
Immunoblotting
225
115
80
65
anti-NgR Ab detecting NgR(310)ecto -myc-Fc
E
IP: pulled down with anti-myc
kDa
Empty vector spleen
NgR-Fc transduced spleen
225
115
80
65
anti-Fc Ab detecting NgR(310)ecto -myc-Fc
F
5% input
kDa
NgR full length peptide
Empty vector spleen
NgR-Fc transduced spleen
225
115
80
65
50
35
anti-NgR Ab detecting NgR(310)ecto -myc-Fc
β-actin

Supplementary Figure 2

Secretion of NgR(310)ecto-Fc fusion protein in the periphery and secondary lymphoid organs.

(**A**) Schematic diagram of non-transduced empty vector-transduced and NgR(310)ecto-Fc-transduced HSCs that are transplanted into irradiated female recipient mice via tail vein injection after 3-hour post-irradiation recovery. (**B**) Schematic diagram of sandwich-ELISA to capture with anti-myc antibody and detect the secretion of NgR(310)ecto-Fc fusion protein in conditioned media, sera and spleen collected from the empty vector and NgR(310)ecto-Fc HSCT animals with primary anti-NgR antibody. (**C**) ELISA determined concentration of NgR(310)ecto-Fc fusion protein in NgR-Fc full length peptide solution (red), empty vector conditioned media (orange), NgR(310)ecto-Fc conditioned media (yellow), empty vector transduced sera (green), NgR(310)ecto-Fc transduced sera (blue) and spleen (purple). One-way ANOVA with Tukey post-hoc test. *$P < 0.05$, **$P < 0.\ 01$, ***$P < 0.001$. Individual data points plotted on the graph represent either pooled media samples obtained from individual experiments following ex vivo HSC transduction, or sera samples and spleen tissue lysates that were collected from individual mice following transplantation of transduced HSCs. (**D-E**) Immunoprecipitation of myc-tagged proteins of the spleen lysates from the empty vector and NgR(310)ecto-Fc HSCT animals, followed by immunoprobing the membranes with (**D**) anti-NgR, and (**E**) anti-Fc antibodies. (**F**) Pre-immunoprecipitation data with 5% input of protein from the spleen lysates collected from the empty- and NgR(310)ecto-Fc-transduced animals, followed by immunoprobing the membranes with anti-NgR and anti-actin antibodies.

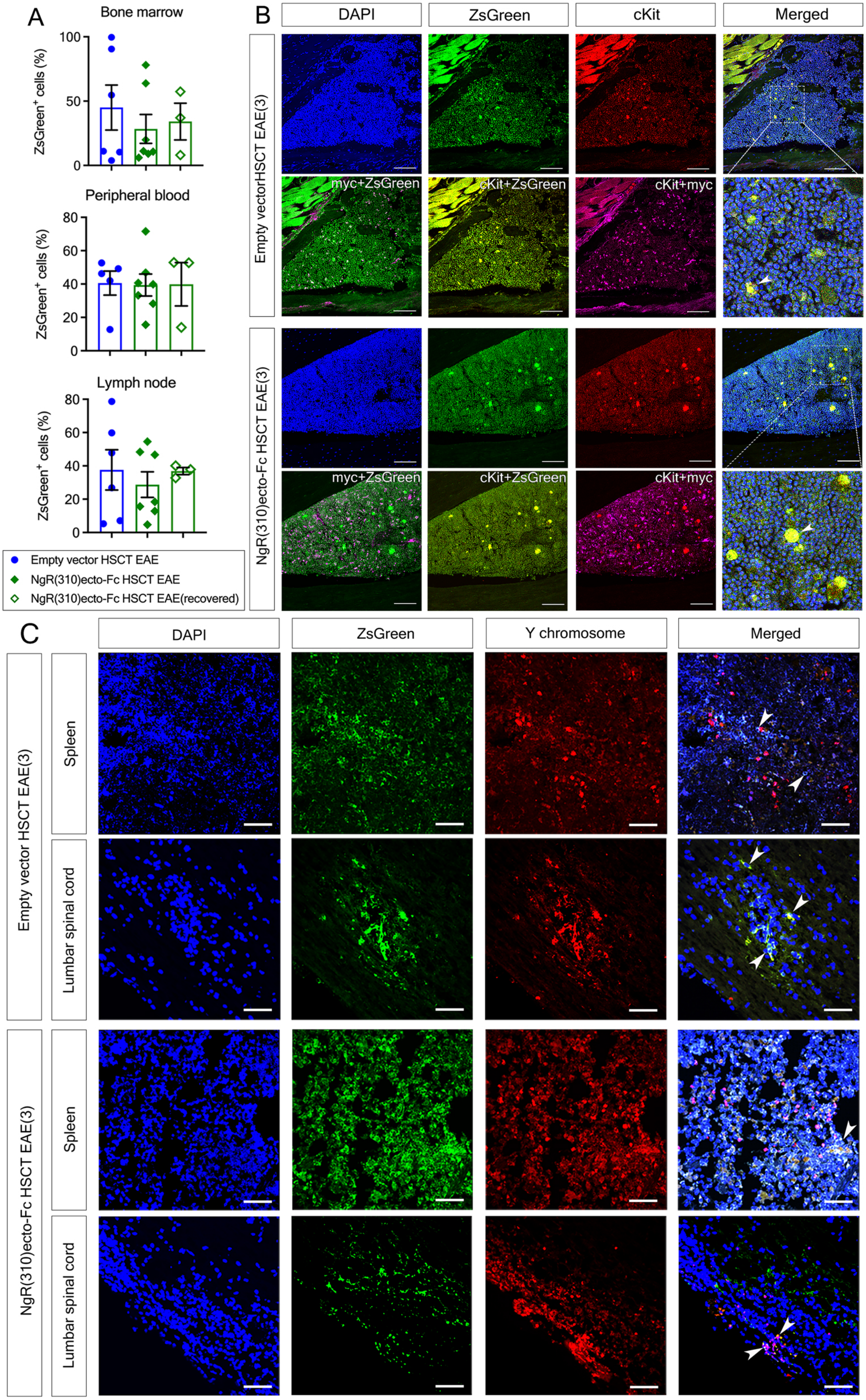
A
Bone marrow
ZsGreen+ cells (%)
100
50
0
Peripheral blood
80
60
40
20
Lymph node
Empty vector HSCT EAE
NgR(310)ecto-Fc HSCT EAE
NgR(310)ecto-Fc HSCT EAE(recovered)
B
DAPI
ZsGreen
cKit
Merged
Empty vectorHSCT EAE(3)
myc+ZsGreen
cKit+ZsGreen
cKit+myc
NgR(310)ecto-Fc HSCT EAE(3)
C
Y chromosome
Empty vector HSCT EAE(3)
Spleen
Lumbar spinal cord
NgR(310)ecto-Fc HSCT EAE(3)

Supplementary Figure 3

ZsGreen expression in peripheral tissues at the peak of disease in lentiviral vector transduced HSC recipient mice.

(**A**) ZsGreen expression was detected in bone marrow, peripheral blood and lymph nodes at the peak stage of disease by flow cytometry. No statistical significant differences were recorded among the groups of empty vector- and NgR(310)ecto-Fc-transduced HSCT at the peak EAE and recovered animals. n = 3-7 per group. Data are represented as mean ± SEM. One-way ANOVA with Tukey post-hoc test. Individual data points represent the analysis of the percentage of ZsGreen+ cells isolated from experimental mice and analysed by flow cytometry. (**B**) Immunohistochemical analysis of ZsGreen (green), cKit (red), and myc (magenta) in bone marrow sections of the empty vector and NgR(310)ecto-Fc HSCT mice at EAE clinical score 3. Lower panel showed the presence of two channels being merged as myc + ZsGreen, cKit + ZsGreen, and cKit + myc. Scale bar = 100 µm. (**C**) Fluorescence in situ hybridization analysis was performed using a Y-chromosome-specific DNA probe to identify male donor HSCs in the spleen and lumbar spinal cords from lentivirally transduced HSC recipient mice by co-localized expression of ZsGreen (green) and Y-chromosome (red) indicated by white arrowheads. Scale bar = 50 µm.

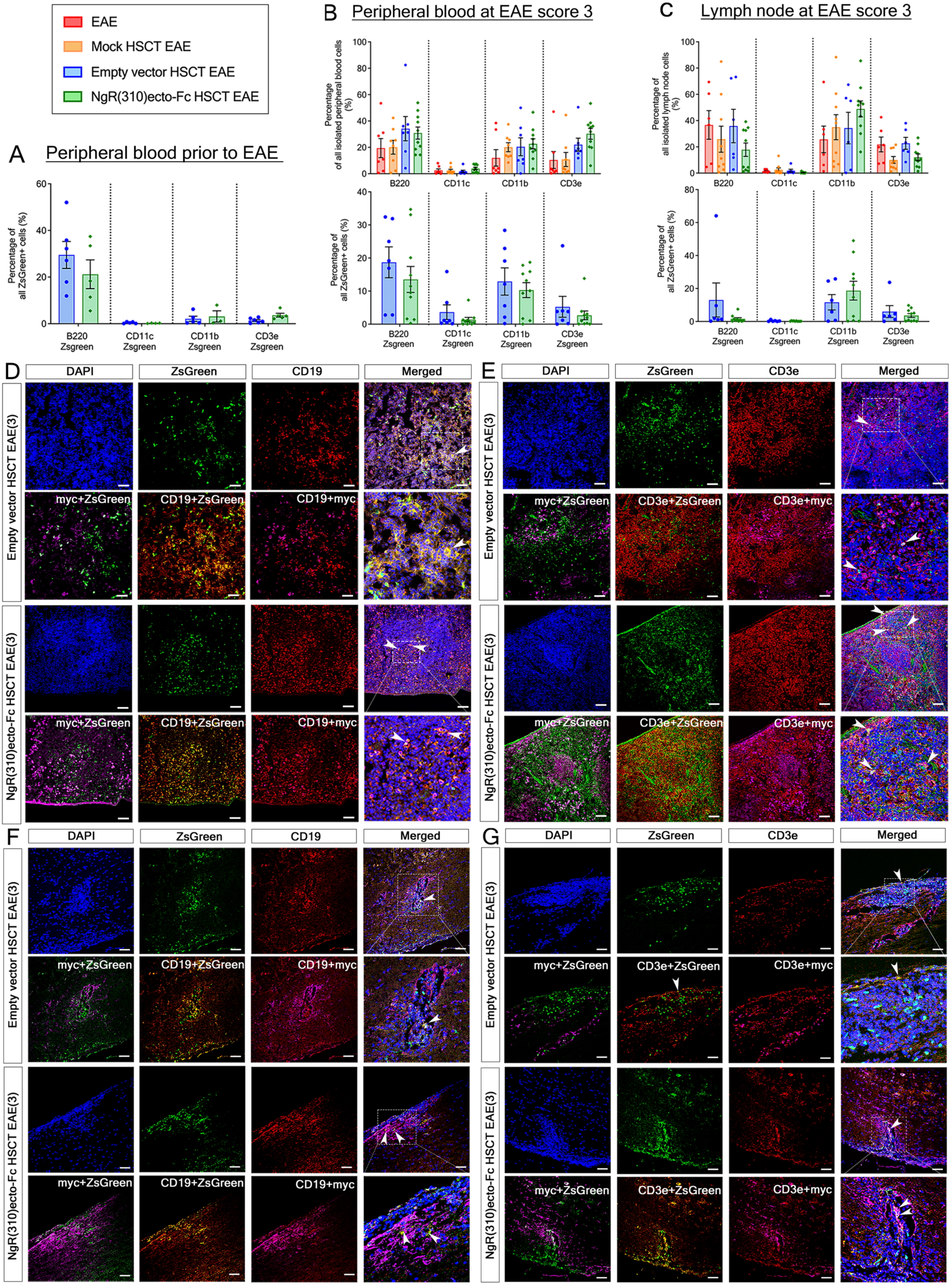

EAE
Mock HSCT EAE
Empty vector HSCT EAE
NgR(310)ecto-Fc HSCT EAE
A
Peripheral blood prior to EAE
Percentage of all ZsGreen+ cells (%)
B220 Zsgreen
CD11c Zsgreen
CD11b Zsgreen
CD3e Zsgreen
B
Peripheral blood at EAE score 3
Percentage of all isolated peripheral blood cells (%)
B220
CD11c
CD11b
CD3e
Percentage of all ZsGreen+ cells (%)
B220 Zsgreen
CD11c Zsgreen
CD11b Zsgreen
CD3e Zsgreen
C
Lymph node at EAE score 3
Percentage of all isolated lymph node cells (%)
B220
CD11c
CD11b
CD3e
Percentage of all ZsGreen+ cells (%)
B220 Zsgreen
CD11c Zsgreen
CD11b Zsgreen
CD3e Zsgreen
D
DAPI
ZsGreen
CD19
Merged
Empty vector HSCT EAE(3)
myc+ZsGreen
CD19+ZsGreen
CD19+myc
NgR(310)ecto-Fc HSCT EAE(3)
E
DAPI
ZsGreen
CD3e
Merged
Empty vector HSCT EAE(3)
myc+ZsGreen
CD3e+ZsGreen
CD3e+myc
NgR(310)ecto-Fc HSCT EAE(3)
F
DAPI
ZsGreen
CD19
Merged
Empty vector HSCT EAE(3)
myc+ZsGreen
CD19+ZsGreen
CD19+myc
NgR(310)ecto-Fc HSCT EAE(3)
G
DAPI
ZsGreen
CD3e
Merged
Empty vector HSCT EAE(3)
myc+ZsGreen
CD3e+ZsGreen
CD3e+myc
NgR(310)ecto-Fc HSCT EAE(3)

Supplementary Figure 4

No significant immunomodulation after lentivirus-transduced HSC transplantation on mature immune cell lineage differentiation.

(**A-C**) Flow cytometric immunophenotyping of the cells. (**A**) Prior to EAE induction, the percentage of ZsGreen+ cells co-expressing either of the B220, CD11c, CD11b, or CD3e surface markers versus the overall percentage of isolated ZsGreen+ cells from the peripheral blood of empty vector and NgR(310)ecto-Fc HSCT mice were determined. (**B-C**) At the peak stage of EAE (clinical score 3), the percentage of ZsGreen+ cells co-expressing either of the B220, CD11c, CD11b, or CD3e surface markers were compared with all lineage cells that were isolated from: the peripheral blood that were ZsGreen+ (**B**) and the lymph nodes that were ZsGreen+ (**C**); were analysed. The level of ZsGreen+ lineage-differentiated immune cells in both empty vector and NgR(310)ecto-Fc vector transduced HSCT recipient mice were similar at the peak stage of disease (EAE clinical score 3). All EAE-induced mice analysed were n = 5-10. Data were represented as mean ± SEM with a two-way ANOVA with Tukey post-hoc test applied. Individual data points plotted in the graph represent isolated cells from tissue samples from experimental mice. (**D-E**) Immunochemical images of CD19+ B lymphocytes (**D**) and CD3e+ T lymphocytes (**E**) in the spleen. Either CD19 or CD3e (red) labelled with ZsGreen (green) and myc (magenta) was demonstrated as transduced HSCs-derived mature immune cells (indicated by arrowheads). Scale bar = 100 µm. (**F-G**) Immunochemical images of ZsGreen lineage CD19+ B (**F**) and CD3e+ T (**G**) immune cells (indicated by arrowhead) at the perivascular area and inflammatory region of spinal cords in the transduced HSCT mice with EAE clinical score 3. Scale bar = 100 µm.

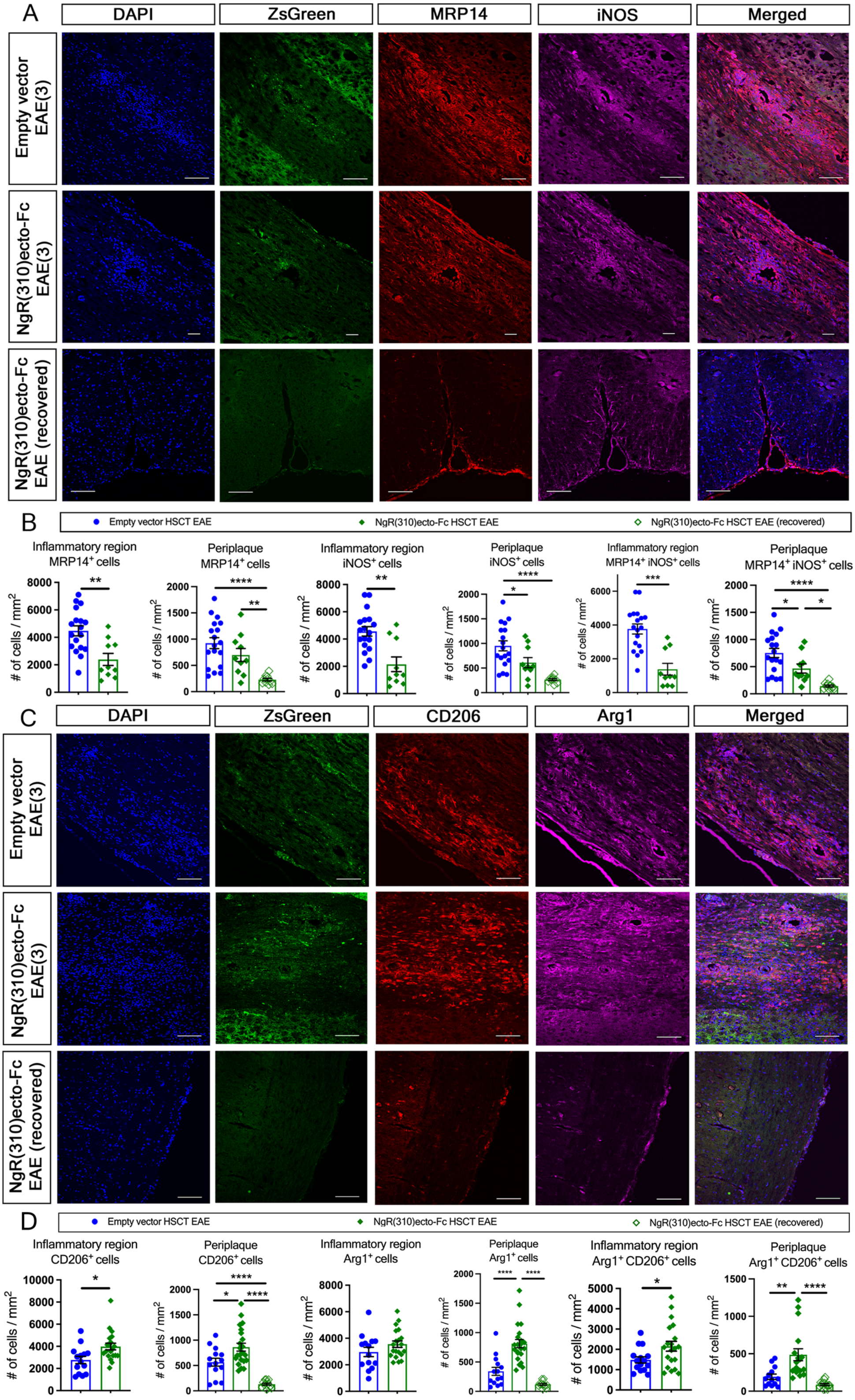

A
DAPI
ZsGreen
MRP14
iNOS
Merged
Empty vector EAE(3)
NgR(310)ecto-Fc EAE(3)
NgR(310)ecto-Fc EAE (recovered)
B
Empty vector HSCT EAE
NgR(310)ecto-Fc HSCT EAE
NgR(310)ecto-Fc HSCT EAE (recovered)
Inflammatory region MRP14+ cells
Periplaque MRP14+ cells
Inflammatory region iNOS+ cells
Periplaque iNOS+ cells
Inflammatory region MRP14+ iNOS+ cells
Periplaque MRP14+ iNOS+ cells
# of cells / mm2
C
DAPI
ZsGreen
CD206
Arg1
Merged
Empty vector EAE(3)
NgR(310)ecto-Fc EAE(3)
NgR(310)ecto-Fc EAE (recovered)
D
Empty vector HSCT EAE
NgR(310)ecto-Fc HSCT EAE
NgR(310)ecto-Fc HSCT EAE (recovered)
Inflammatory region CD206+ cells
Periplaque CD206+ cells
Inflammatory region Arg1+ cells
Periplaque Arg1+ cells
Inflammatory region Arg1+ CD206+ cells
Periplaque Arg1+ CD206+ cells
# of cells / mm2

Supplementary Figure 5

Phenotyping the endogenous microglia and macrophage populations during EAE progression in empty and NgR(310)ecto-Fc vector administered HSCT mice.

(**A**) Immunochemical staining of proinflammatory ZsGreen+ (green) macrophages labelled with MRP14 (red) and iNOS (magenta) within the spinal cord white matter inflammatory lesions identified in empty- and NgR(310)ecto-Fc-transduced HSCT recipient mice that either exhibited an EAE clinical score 3 or were clinically recovered. Scale bar = 100 µm. (**B**) The calculated number of cells per unit area of the inflammatory regions (IR) and peri-plaque white matter (PPWM) areas of spinal cord are demonstrated for MRP14+ cells, iNOS+ cells, and MRP14+iNOS+ double labelled cells (n = 3 per group). Data were represented as mean ± SEM. Mann-Whitney two-tailed t-test. Unpaired two-tailed t-test, One-way ANOVA with Tukey post-hoc test. **P* < 0.05, ***P* < 0. 01, ****P* < 0.001, *****P* < 0.0001. Individual data points represent the cell numbers obtained from inflammatory and non-inflammatory regions of spinal cord (longitudinal sections) of experimental mice. (**C**) Immunochemical staining of ZsGreen+ (green) macrophages labelled with CD206 (red) and Arg1 (magenta) in the spinal cord of empty vector, NgR(310)ecto-Fc HSCT recipients at the peak EAE clinical score 3 and recovered NgR(310)ecto-Fc HSCT. Scale bar = 100 µm. (**D**) The calculated number of cells per unit area of the IR and PPWM areas of spinal cord are demonstrated for CD206+ cells, Arg1+ cells, and CD206+Arg1+ double labelled cells (n = 3 per group). Data were represented as mean ± SEM. Unpaired two-tailed t-test, One-way ANOVA with Tukey post-hoc test. **P* < 0.05, ***P* < 0. 01, *****P* < 0.0001. Individual data points represent the cell numbers obtained from inflammatory and non-inflammatory regions of spinal cord (longitudinal sections) of experimental mice.

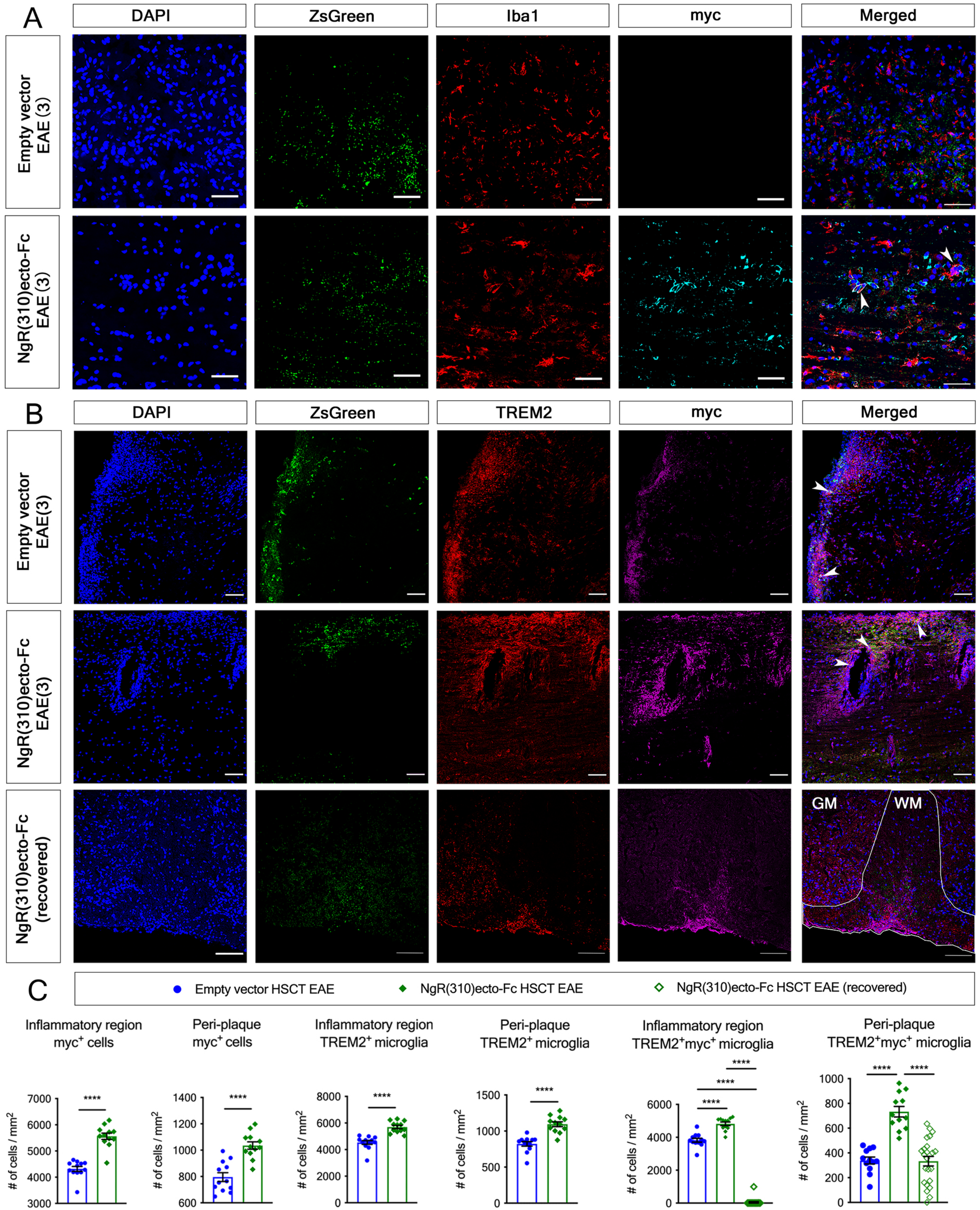

A
DAPI
ZsGreen
Iba1
myc
Merged
Empty vector EAE (3)
NgR(310)ecto-Fc EAE (3)
B
DAPI
ZsGreen
TREM2
myc
Merged
Empty vector EAE(3)
NgR(310)ecto-Fc EAE(3)
NgR(310)ecto-Fc (recovered)
GM
WM
C
Empty vector HSCT EAE
NgR(310)ecto-Fc HSCT EAE
NgR(310)ecto-Fc HSCT EAE (recovered)
Inflammatory region myc+ cells
Peri-plaque myc+ cells
Inflammatory region TREM2+ microglia
Peri-plaque TREM2+ microglia
Inflammatory region TREM2+myc+ microglia
Peri-plaque TREM2+myc+ microglia
# of cells / mm2
****

Supplementary Figure 6

Phenotyping the endogenous microglia and macrophage populations during EAE progression in empty and NgR(310)ecto-Fc vector administered HSCT mice.

(**E**) Immunochemical staining of microglia labelled with Iba1 (red) and myc (magenta) within the inflammatory lesion in the spinal cords of the empty vector- and NgR(310)ecto-Fc-transduced HSCT recipient with EAE clinical score 3. Scale bar = 50 μm. (**F**) Immunochemical staining of ZsGreen (green) colocalized with the homeostatic microglia marker TREM2 (red) and myc (magenta) was conducted in the spinal cord of empty vector, NgR(310)ecto-Fc HSCT recipients at the peak EAE clinical score 3 and recovered NgR(310)ecto-Fc HSCT. Scale bar = 100 μm. (**G**) The calculation of myc+ cells, TREM2+ microglia and colocalized TREM2+myc+ microglia number per the IR and PPWM area of spinal cords in either the empty or NgR(310)ecto-Fc HSCT recipient mice at the peak stage and clinically recovered NgR(310)ecto-Fc group. TREM2+myc+ microglial cell numbers per IR and PPWM area in mice carrying the NgR(310)ecto-Fc vector at the peak stage of the disease significantly increased when compared with the mice containing the empty vector. n = 3 per group. Data were represented as mean ± SEM. Mann-Whitney two-tailed t-test. One-way ANOVA with Tukey post-hoc test. ****P < 0.0001. Individual data points represent the cell numbers obtained from inflammatory and non-inflammatory regions of spinal cord (longitudinal sections) of experimental mice.

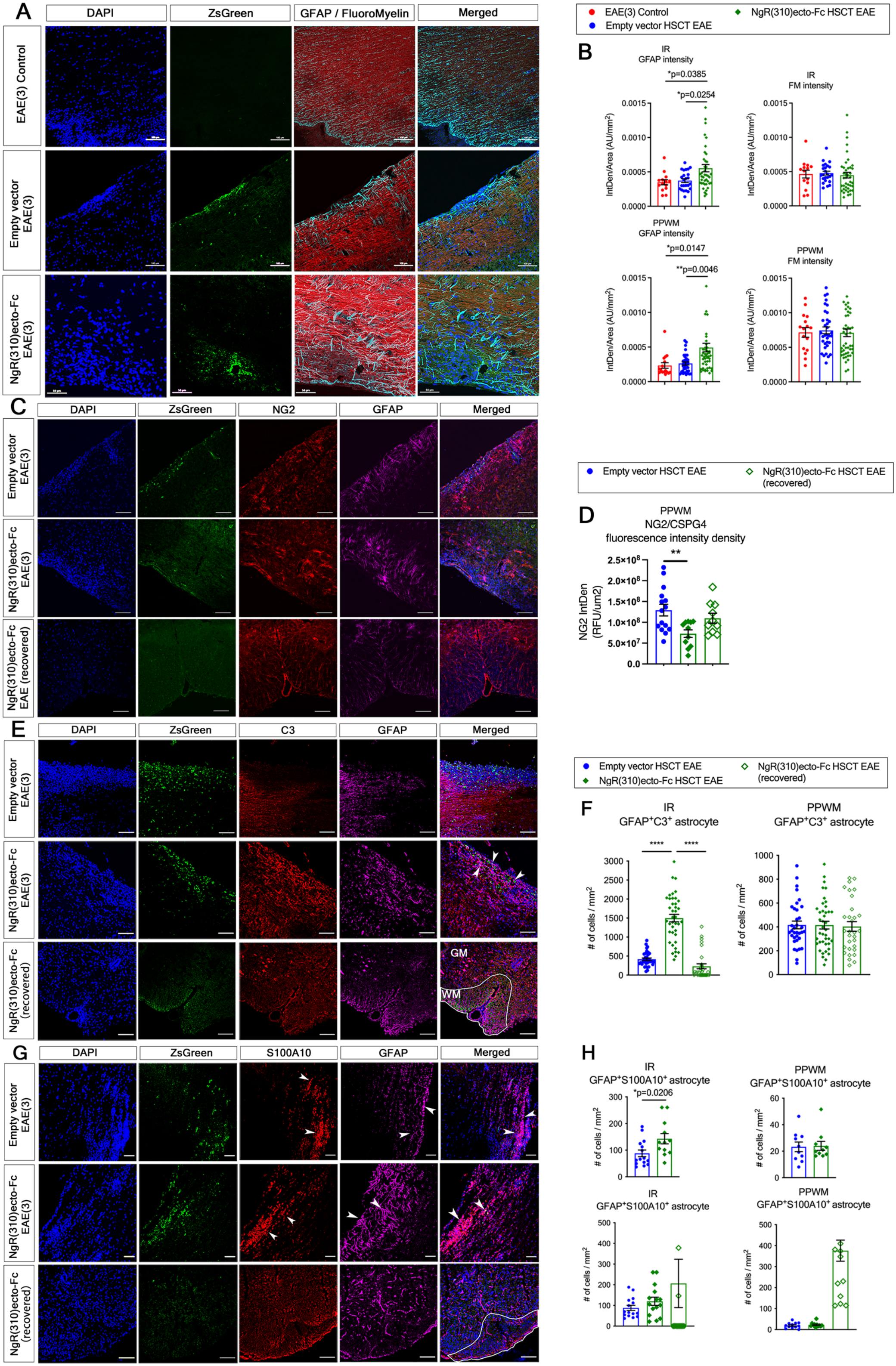

A
DAPI
ZsGreen
GFAP / FluoroMyelin
Merged
EAE(3) Control
Empty vector EAE(3)
NgR(310)ecto-Fc EAE(3)
EAE(3) Control
NgR(310)ecto-Fc HSCT EAE
Empty vector HSCT EAE
B
IR GFAP intensity
*p=0.0385
*p=0.0254
IR FM intensity
PPWM GFAP intensity
*p=0.0147
**p=0.0046
PPWM FM intensity
IntDen/Area (AU/mm²)
C
DAPI
ZsGreen
NG2
GFAP
Merged
Empty vector EAE(3)
NgR(310)ecto-Fc EAE(3)
NgR(310)ecto-Fc EAE (recovered)
Empty vector HSCT EAE
NgR(310)ecto-Fc HSCT EAE (recovered)
D
PPWM NG2/CSPG4 fluorescence intensity density
**
NG2 IntDen (RFU/um2)
E
DAPI
ZsGreen
C3
GFAP
Merged
Empty vector EAE(3)
NgR(310)ecto-Fc EAE(3)
NgR(310)ecto-Fc (recovered)
GM
WM
Empty vector HSCT EAE
NgR(310)ecto-Fc HSCT EAE
NgR(310)ecto-Fc HSCT EAE (recovered)
F
IR GFAP+C3+ astrocyte
****
****
PPWM GFAP+C3+ astrocyte
# of cells / mm²
G
DAPI
ZsGreen
S100A10
GFAP
Merged
Empty vector EAE(3)
NgR(310)ecto-Fc EAE(3)
NgR(310)ecto-Fc (recovered)
H
IR GFAP+S100A10+ astrocyte
*p=0.0206
PPWM GFAP+S100A10+ astrocyte
IR GFAP+S100A10+ astrocyte
PPWM GFAP+S100A10+ astrocyte
# of cells / mm²

Supplementary Figure 7

Phenotyping the populations of astrocytes and quantifying the expression of CSPG4+ extracellular matrix labelling during EAE progression in empty and NgR(310)ecto-Fc vector-administered HSCT recipient mice.

(**A**) Astrocytes were identified by the colocalization of the universal astrocytic cell marker GFAP (cyan) and FluoroMyelin (red). Labelling was identified within the lesioned demyelinated regions of the spinal cord of either the EAE-induced control, empty vector and NgR(310)ecto-Fc HSCT recipient mice at the peak stage of EAE (clinical score 3). (**B**) The quantification of GFAP+ and FluoroMyelin+ fluorescence intensity per IR and PPWM area was plotted. Elevated numbers of astrocytes within the spinal cords of NgR(310)ecto-Fc HSCT mice are located near demyelinated axons when compared with empty vector HSCT recipient mice. Data were represented as mean ± SEM. One-way ANOVA with Tukey post-hoc test. *$P < 0.05$, **$P < 0.001$. Individual data points represent the analysis of fluorescence intensity density obtained from the longitudinal spinal cord sections of experimental mice. (**C**) Astrocytic extracellular deposits were identified by fluorescence labelling of GFAP (magenta) and CSPG4 (red) within the lesional demyelinated regions of the spinal cords of either empty vector and NgR(310)ecto-Fc HSCT recipient at the peak stage of EAE (clinical score 3) or clinically recovered NgR(310)ecto-Fc HSCT recipient mice. (**D**) The fluorescence intensity density of CSPG4+ labelling per PPWM area was shown in empty vector and NgR(310)ecto-Fc HSCT recipients at the peak stage and recovery stage following EAE. Data were represented as mean ± SEM. One-way ANOVA with Tukey post-hoc test. **$P < 0.001$. Individual data points represent the analysis of fluorescence intensity density obtained from the longitudinal spinal cord sections of experimental mice. (**E-H**) Immunohistochemical staining of astrocytes (GFAP) co-expressed with either C3 (red; **E**) or S100A10 (red; **G**) determined the respective populations of either the Astrocyte phenotype 1 (A1) or Astrocyte phenotype 2 (A2). The

analysis of A1 (**F**) and A2 (**H**) cell numbers per IR and PPWM area in the empty vector, NgR(310)ecto-Fc HSCT mice at the peak stage of the disease (EAE clinical score 3) and the NgR(310)ecto-Fc HSCT recipient mice at the recovered stage is represented. Reactive astrocytes localised within inflammatory demyelinating lesions represented a phenotypic switch of astrocytes in NgR(310)ecto-Fc HSCT recipient mice following their recover from EAE. Scale bar = 100 μm. n = 3 per group. Data were represented as mean ± SEM. Mann-Whitney two-tailed t-test. One-way ANOVA with Tukey post-hoc test. ****P < 0.0001. Individual data points represent the cell numbers obtained from the longitudinal spinal cord sections of experimental mice.

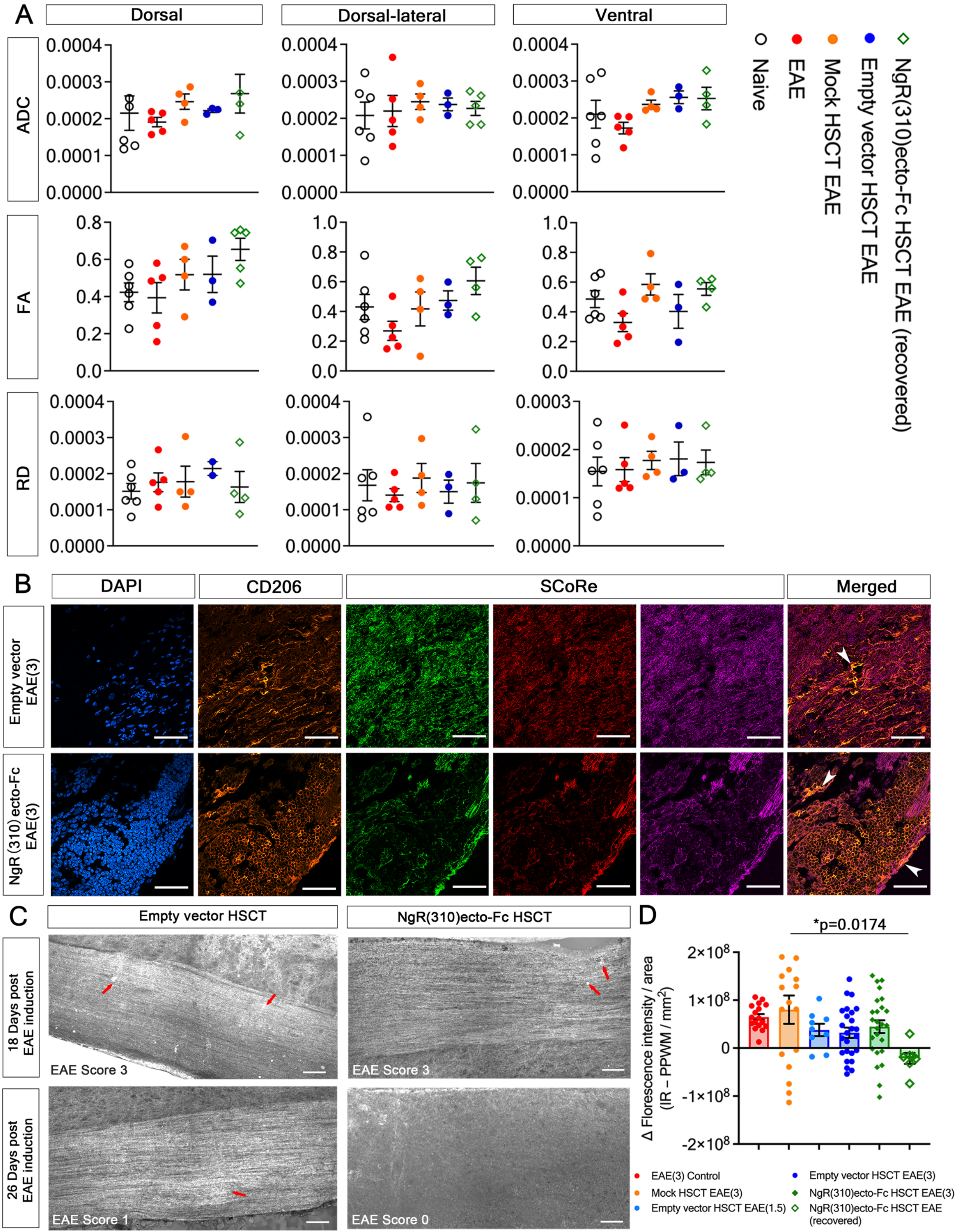

A
Dorsal
Dorsal-lateral
Ventral
ADC
FA
RD
Naive
EAE
Mock HSCT EAE
Empty vector HSCT EAE
NgR(310)ecto-Fc HSCT EAE (recovered)
B
DAPI
CD206
SCoRe
Merged
Empty vector EAE(3)
NgR (310) ecto-Fc EAE(3)
C
Empty vector HSCT
NgR(310)ecto-Fc HSCT
18 Days post EAE induction
26 Days post EAE induction
EAE Score 3
EAE Score 3
EAE Score 1
EAE Score 0
D
*p=0.0174
Δ Florescence intensity / area (IR – PPWM / mm²)
EAE(3) Control
Mock HSCT EAE(3)
Empty vector HSCT EAE(1.5)
Empty vector HSCT EAE(3)
NgR(310)ecto-Fc HSCT EAE(3)
NgR(310)ecto-Fc HSCT EAE (recovered)

Supplementary Figure 8

Diminished SCoRe of myelin debris observed in the inflammatory region of regenerated spinal cords from mice administered with lentiviral-transduced NgR(310)ecto-Fc vector. (**A**) ADC, FA and RD measurements of dorsal, dorsal-lateral and ventral tract in naïve, EAE control, Mock HSCT EAE, empty vector-transduced and clinically recovered NgR(310)ecto-Fc HSCT mice. n = 3-6. One-way ANOVA and Tukey post-hoc test. **P* < 0.05, ***P* < 0.001, *****P* < 0.0001. (**B**) Representative Images of SCoRe with immunostaining of CD206+ (yellow) macrophage engulfing myelin debris (green, red and magenta) in inflammatory region in mice transplanted with NgR(310)-ecto-Fc HSCs (indicated by arrowheads). Scale bar = 50μm. (**C**) Reflective microscopy indicated compact myelin expression and myelin debris (red arrowheads) in empty vector- and NgR(310)ecto-Fc-overexpressed cords collected from post-transduction Day 18 (the peak stage of EAE; clinical score 3) and Day 26 (the recovered stage of EAE; clinical score 0). These compact myelinated axons present SCoRe as an elongated and continuous pattern of line. However, the inflammatory region involving demyelination and immune cells accumulation results in the lack of SCoRe, their morphology was shown as patches of intense reflective detritus. (**D**) Myelin debris reflectance in IR minus myelin debris reflectance in PPWM were calculated, demonstrating the dramatic reduction of myelin debris reflectance in the inflammatory lesion cords during the repair phase of EAE in the NgR(310)ecto-Fc HSCT mice. n = 1 in empty vector HSCT EAE (1.5), n = 4 in other analyzed groups. Data were represented as mean ± SEM. Two-way ANOVA with Tukey post-hoc test. **P* < 0.05. Individual data points represent the analysis of fluorescence intensity obtained from the longitudinal spinal cord sections of experimental mice.

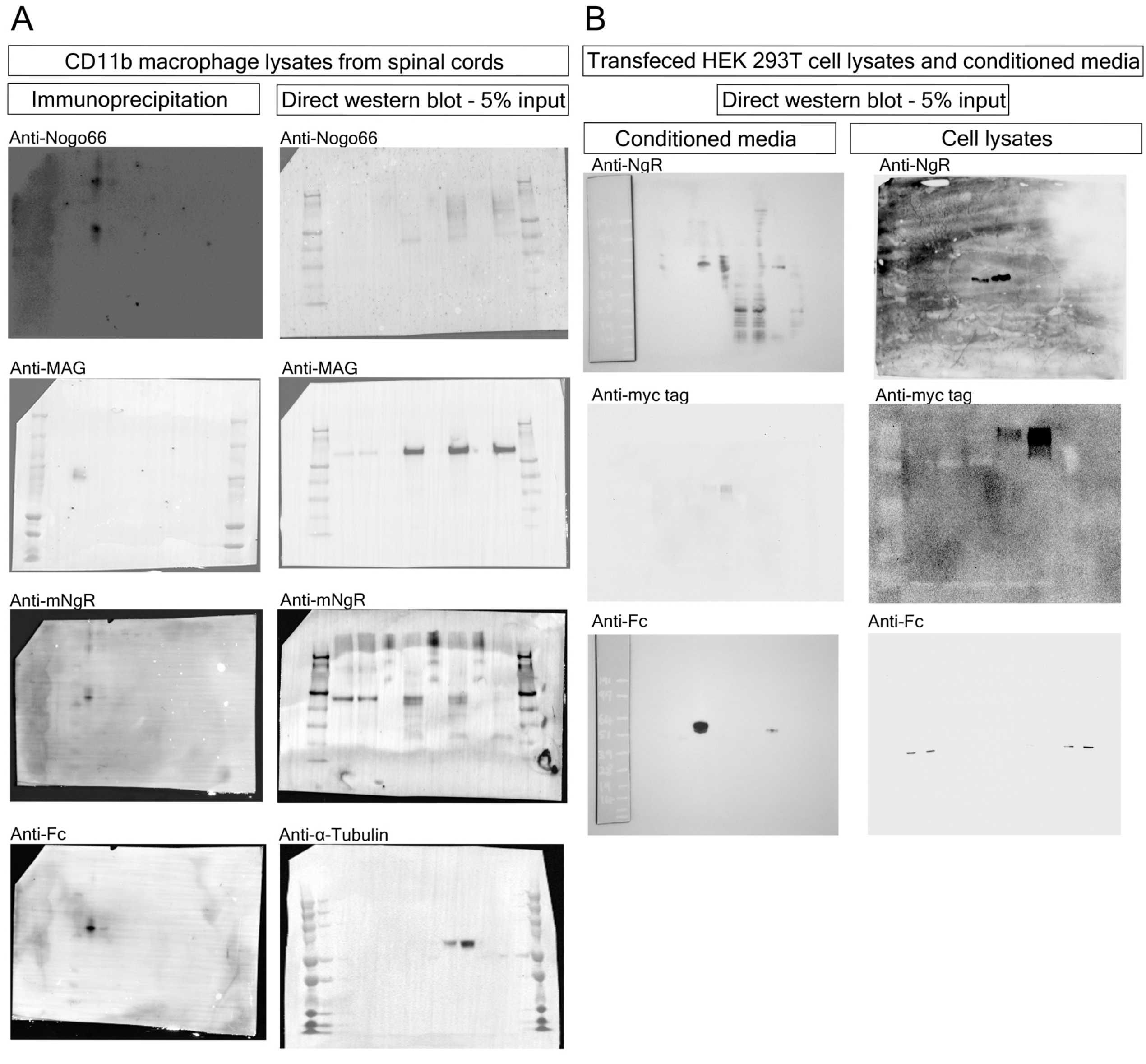
A
CD11b macrophage lysates from spinal cords
Immunoprecipitation
Direct western blot - 5% input
Anti-Nogo66
Anti-Nogo66
Anti-MAG
Anti-MAG
Anti-mNgR
Anti-mNgR
Anti-Fc
Anti-α-Tubulin
B
Transfeced HEK 293T cell lysates and conditioned media
Direct western blot - 5% input
Conditioned media
Cell lysates
Anti-NgR
Anti-NgR
Anti-myc tag
Anti-myc tag
Anti-Fc
Anti-Fc

Supplementary Figure 9

Uncropped blot scans for the presented immunoprecipitation and western blots. (**A**) corresponds to Fig. 5B; (**B**) corresponds to Supplementary Figure 1B.
